# Selective Nitrate Recognition by a Halogen‐Bonding Four‐Station [3]Rotaxane Molecular Shuttle

**DOI:** 10.1002/anie.201604327

**Published:** 2016-07-20

**Authors:** Timothy A. Barendt, Andrew Docker, Igor Marques, Vítor Félix, Paul D. Beer

**Affiliations:** ^1^ Chemistry Research LaboratoryDepartment of ChemistryUniversity of Oxford12 Mansfield RoadOxfordOX1 3TAUK; ^2^ Department of ChemistryCICECO—Aveiro Institute of MaterialsDepartment of Medical SciencesiBiMED—Institute of BiomedicineUniversity of Aveiro3810-193AveiroPortugal

**Keywords:** halogen bonding, molecular devices, molecular dynamics, rotaxanes, supramolecular chemistry

## Abstract

The synthesis of the first halogen bonding [3]rotaxane host system containing a bis‐iodo triazolium‐bis‐naphthalene diimide four station axle component is reported. Proton NMR anion binding titration experiments revealed the halogen bonding rotaxane is selective for nitrate over the more basic acetate, hydrogen carbonate and dihydrogen phosphate oxoanions and chloride, and exhibits enhanced recognition of anions relative to a hydrogen bonding analogue. This elaborate interlocked anion receptor functions via a novel dynamic pincer mechanism where upon nitrate anion binding, both macrocycles shuttle from the naphthalene diimide stations at the periphery of the axle to the central halogen bonding iodo‐triazolium station anion recognition sites to form a unique 1:1 stoichiometric nitrate anion–rotaxane sandwich complex. Molecular dynamics simulations carried out on the nitrate and chloride halogen bonding [3]rotaxane complexes corroborate the ^1^H NMR anion binding results.

The prevalence of negatively charged species in biology and in the environment has deemed the development of synthetic anion receptors capable of their strong and selective recognition an important field of chemical research.^1^, ^2^, ^3^ The nitrate anion in particular is an environmental pollutant when leeched into lakes and rivers resulting from anthropogenic overuse of fertilizers or by acid rain.^4^ Medically, an over exposure to nitrate via contaminated drinking water is associated with the formation of carcinogenic nitrosamines and a range of diseases such as methemoglobinemia (blue baby syndrome) in infants.^5^

The design of synthetic receptors capable of the selective recognition of nitrate is a challenge because of the anion's inherent low affinity for hydrogen bonds and high energy of solvation.^6^ To date only a relatively small number of nitrate‐selective tripodal acyclic, macrocyclic and cage‐like host systems have been reported that utilise convergent hydrogen bonding (HB) and/or anion–π interactions to recognise the oxoanion in polar organic solvents.^7^, ^8^, ^9^, ^10^, ^11^, ^12^, ^13^, ^14^, ^15^, ^16^, ^17^, ^18^, ^19^

The challenge of developing synthetic receptors that can rival the anion recognition properties of biotic systems relies upon the arrangement of a three‐dimensional convergent array of numerous HB donor groups in an optimized geometry for recognition of the complementary guest.^20^, ^21^ To meet this challenge we have exploited anion‐templation to construct interlocked host structures^22^, ^23^ whose unique three‐dimensional cavities encapsulate anionic guest species.

While during the past two decades HB has been widely exploited in anion receptor design, halogen bonding (XB),^24^, ^25^, ^26^, ^27^ the attractive highly directional interaction between an electron‐deficient halogen atom and a Lewis base, has only recently begun to be utilised for anion recognition. Of the relatively few examples of XB anion receptors reported to date, it is noteworthy that all display promising, and significantly contrasting, anion recognition behaviour when compared to HB analogues, by virtue of their comparable bond strengths and more strict linear geometry preference.^28^, ^29^, ^30^, ^31^, ^32^, ^33^, ^34^, ^35^, ^36^, ^37^, ^38^, ^39^, ^40^, ^41^, ^42^ Importantly in a significant step forward for highlighting the potential importance of halogen bonding in anion supramolecular chemistry, we have recently demonstrated the first examples of solution phase halogen bonding being exploited to control and facilitate the anion‐templated assembly of interlocked structures^43^, ^44^, ^45^, ^46^, ^47^ and demonstrated that the incorporation of halogen bond donor atoms into a [2]rotaxane host cavity dramatically improves the anion recognition capabilities of the XB interlocked receptor.^48^, ^49^, ^50^, ^51^

Herein, we report the synthesis of the first halogen bonding [3]rotaxane host system, containing a bis‐iodo triazolium‐bis‐naphthalene diimide four station axle component, which employs multiple cooperative XB and HB interactions to exhibit enhanced recognition of anions relative to an all‐HB analogue and, impressively, is found to be selective for nitrate over other oxoanions and chloride. Integrated into the extremities of the axle component of the [3]rotaxane are two electron deficient naphthalene diimide (NDI) groups that act as recognition sites (or stations) for each macrocycle component in the absence of a coordinating anion by donor–acceptor charge‐transfer interactions. This produces an exotic anion receptor that functions via a novel dynamic pincer mechanism in which both macrocycles shuttle from the periphery of the axle component towards the central halogen bonding bis‐iodo‐triazolium recognition sites to form a unique 1:1 stoichiometric nitrate anion‐rotaxane sandwich complex (Figure 1). Therefore, this adaptable receptor is also an example of a complex molecular switch, since it produces reversible pincer‐like molecular motion, through changes in the relative positions of the macrocycle components at the recognition sites on the axle upon exposure to anionic chemical stimuli.

**Figure 1:**
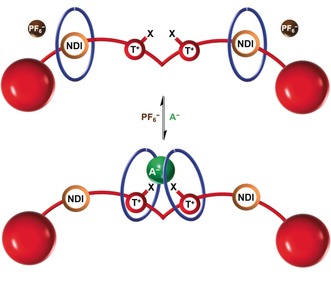
Schematic of halogen bonding [3]rotaxane host design for the recognition of oxoanions (e.g. A^−^=NO_3_^−^) via a dynamic molecular shuttling mechanism upon anion recognition. NDI=naphthalene diimide, T^+^=triazolium and X=I or H.

The dicationic HB and XB axle components **1⋅(Cl)_2_** and **2⋅(Cl)_2_** were synthesised using a multistep procedure shown in Scheme S1 in the Supporting Information (SI), incorporating two NDI motifs, as macrocycle stations, each separated from the central bis‐triazolium anion recognition site by a rigid biphenyl spacer unit.

Rotaxane syntheses were undertaken by a chloride anion‐templated clipping methodology (Scheme 1). The reaction of two equivalents of bis‐vinyl‐functionalised macrocyclic precursor **3** with the appropriate HB, **1⋅(Cl)_2_** or XB, **2⋅(Cl)_2_** axle in the presence of Grubbs’ II catalyst in dry dichloromethane afforded, after purification by preparative silica thin‐layer chromatography, the desired HB and XB [3]rotaxanes, **4⋅(Cl)_2_** and **5⋅(Cl)_2_** respectively (Scheme 1).

**Scheme 1:**
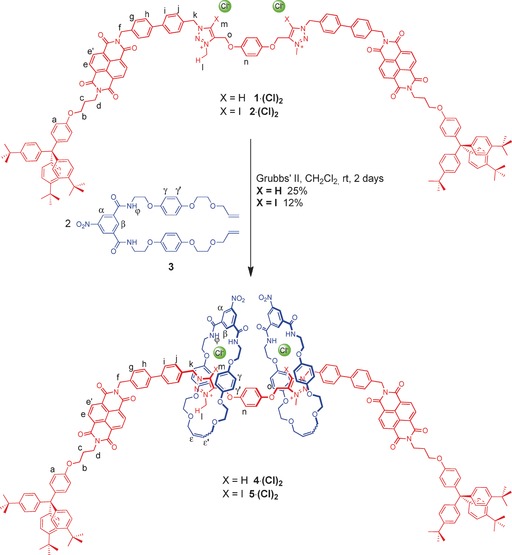
Synthesis of the HB and XB multi‐station [3]rotaxanes **4⋅(Cl)_2_** and **5⋅(Cl)_2_**, indicating the predicted co‐conformation of the molecules as their chloride salts.

Both rotaxanes were characterised by ^1^H and ^13^C NMR spectroscopy and by high resolution mass spectrometry (see SI). The interlocked nature of the rotaxane species was confirmed by two‐dimensional ^1^H ROESY NMR spectroscopy which also provided evidence for the co‐conformations depicted in Scheme 1. Cross coupling correlations between the mechanically bonded macrocycle (H_γ,γ′_ and H_*ϵ*,*ϵ*′_) and diagnostic protons of the bis triazolium station of the axle components (H_n_, H_k,_ H_l_ and H_o_) indicate both macrocycles symmetrically occupying the central recognition site courtesy of convergent HB and/or XB to each of the chloride counter anions (Figures S1 and S2). An absence of cross coupling between the macrocycle and NDI station protons (Figures S1 and S2) and small differences between *δ*(H_e,e′_) in the ^1^H NMR spectra of [3]rotaxanes **4⋅(Cl)_2_** and **5⋅(Cl)_2_** and the bis‐chloride salts of their corresponding axles indicates there are negligible interactions between macrocycles and the NDI stations (Figures S3 and S4).

Anion exchange to the corresponding PF_6_^−^ salts was achieved by repeatedly passing a solution of the rotaxane chloride salt through an Amberlite ion exchange column. A comparison of the resulting ^1^H NMR spectra in CDCl_3_ of rotaxanes **4⋅(PF_6_)_2_** and **5⋅(PF_6_)_2_** with their respective chloride salts **4⋅(Cl)_2_** and **5⋅(Cl)_2_** indicates a change in the co‐conformation has occurred as a result of shuttling of the macrocycle components to symmetrically occupy the peripheral NDI stations of the axle (Figures 2 and S5). This is inferred by significant upfield shifts of the macrocycle hydroquinone protons H_γ,γ′_, indicative of stronger aromatic donor–acceptor charge‐transfer interactions between the axle's electron deficient NDI and macrocycle hydroquinone motifs. The changes to the co‐conformations are further evidenced by the two‐dimensional ^1^H ROESY NMR spectra of **4⋅(PF_6_)_2_** and **5⋅(PF_6_)_2_**, that both show the appearance of new correlations between resonance signals arising from the macrocycle protons H_γ,γ′_ and H_*ϵ*,*ϵ*′_ with the NDI protons H_e,e′_ (Figures S6 and S7). A comparison of the ^1^H NMR spectra of **4⋅(PF_6_)_2_** and **5⋅(PF_6_)_2_** with the bis‐hexafluorophosphate salts of their respective axle components also revealed the macrocycle components interact significantly with the NDI stations (Figures S8 and S9).

**Figure 2:**
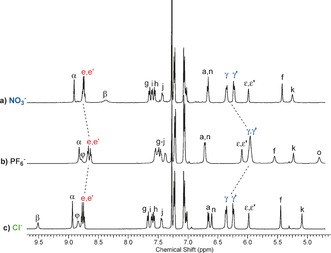
Comparison of the truncated ^1^H NMR spectra (CDCl_3_, 298 K, 400 MHz) of XB [3]rotaxanes a) **5⋅(NO_3_)⋅(PF_6_)**, b) **5⋅(PF_6_)_2_** and c) **5⋅(Cl)_2_**.

To test the ability of the HB and XB [3]rotaxanes to behave as translational molecular shuttles stimulated by the chemical recognition of an oxoanion such as nitrate, one equivalent of (tetrabutylammonium)NO_3_ was added to the rotaxanes **4⋅(PF_6_)_2_** and **5⋅(PF_6_)_2_** in CDCl_3_ solution. The resulting ^1^H NMR spectra are almost identical to that of their respective chloride salts indicating that both systems undergo a concerted molecular pincer motion of the macrocycles from the peripheral NDI stations into the centre of the axle to participate in binding of the nitrate anion at the bis‐triazolium station via formation of a 1:1 sandwich complex (Figures 2 and S5). This change in co‐conformation of both [3]rotaxane systems was confirmed using two‐dimensional ^1^H ROESY NMR which indicated the re‐emergence of cross coupling between the macrocycle protons H_γ,γ′_ and H_*ϵ*,*ϵ*′_ and the axle protons associated with the bis‐triazolium station (Figures S10 and S11). Importantly the spectra show no cross coupling interactions between the macrocycle hydroquinone protons H_γ,γ′_ and the NDI aromatic protons H_e,e′_ implying that any interactions with this station prior to nitrate addition have since been reduced. The same conclusion can also be drawn from a comparison of the ^1^H NMR spectra of **4⋅(NO_3_)⋅(PF_6_)** and **5⋅(NO_3_)⋅(PF_6_)** with their respective axle components (Figures S3 and S4). The operational cycle of the [3]rotaxane molecular shuttles is completed upon anion exchange of **4⋅(NO_3_)⋅(PF_6_)** and **5⋅(NO_3_)⋅(PF_6_)** back to their bis‐hexafluorophosphate salts using NaPF_6(s)_ (Figures S5 and S12).

Additional evidence for the dynamic behaviour exhibited by these systems comes from a “naked eye” colour change of the [3]rotaxane in CHCl_3_ that occurs on addition of a coordinating anion (Figure 3). As the non‐coordinating PF_6_^−^ salt the [3]rotaxanes produce a strongly coloured orange solution courtesy of the donor–acceptor charge‐transfer interactions that dominate between the NDI stations of the axle and the hydroquinone motifs of the macrocycle components. Upon addition of a coordinating anion, the solution becomes colourless indicating that the macrocycles have moved away from the NDI groups and occupy the central bis‐triazolium anion recognition sites resulting in a loss of the charge‐transfer interactions, an effect that can be easily monitored by UV/Vis spectroscopy (Figure S13). This means that these [3]rotaxane host systems have the propensity to act as optical sensors for anions by exploiting the novel dynamic behaviour of their constituent parts stimulated by anion recognition.

**Figure 3:**
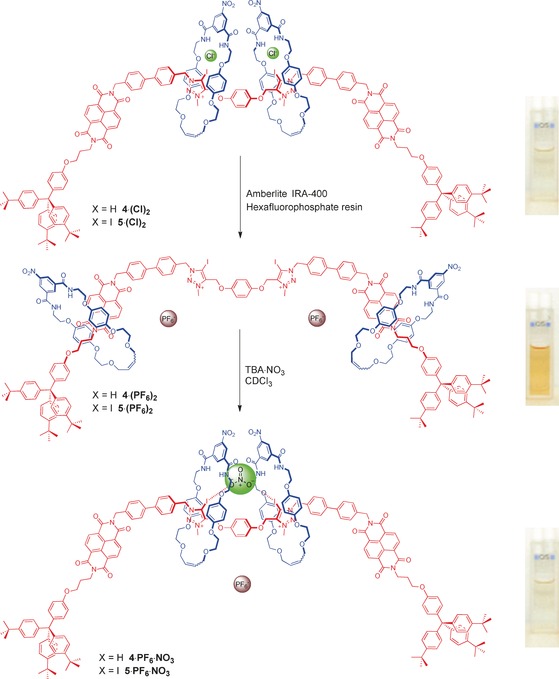
Co‐conformations of XB [3]rotaxanes **5⋅(Cl)_2_**, **5⋅(PF_6_)_2_** and**⋅5⋅(NO_3_)⋅(PF_6_)** and photographs showing the “naked eye” colour changes.

The anion binding properties of the [3]rotaxanes were investigated by ^1^H NMR titration experiments in the competitive solvent mixture of 1:1 CDCl_3_:CD_3_OD. Upon the addition of anions to the respective rotaxane bis‐hexafluorophosphate salts, significant downfield chemical shift perturbations of the methylene protons H_o_ adjacent to the triazolium recognition sites were observed as well as similar changes to the macrocycle's acidic internal isophthalamide proton H_β_ (Figures S14 to S22). These proton perturbations are diagnostic of anion binding occurring at the charged triazolium stations aided by hydrogen bonding interactions from the macrocycles to achieve encapsulation of the anion within the unique cavity created by the three components as shown in Figure 3. WinEQNMR2 analysis of the titration data,^52^ monitoring either protons H_o_ or H_β_, enabled the determination of the anion association constants shown in Table 1. Importantly all were found to exhibit 1:1 stoichiometry^53^, ^54^ apart from chloride (1:2 host:guest model), presumably because of the propensity for the larger oxoanions to bridge the bis‐triazolium recognition site and to form a sandwich complex via favourable HB interactions between the macrocycle components.

**Table 1:** Anion association constants *K*_Ass_ (M^−1^) for rotaxanes **4⋅(PF_6_)_2_** and **5⋅(PF_6_)_2_** determined by ^1^H NMR spectroscopy in 1:1 CDCl_3_:CD_3_OD using chemical shift data of H_o_ unless otherwise stated (298 K, 500 MHz). Anions added as tetrabutylammonium salts unless otherwise stated. Errors <10 %.

Anion	HB Rotaxane (**4⋅(PF_6_)_2_**) *K*_Ass_	XB Rotaxane (**5⋅(PF_6_)_2_**) *K*_Ass_
Cl^−^	507^[a]^	1357^[a]^
NO_3_^−^	653	1782
H_2_PO_4_^−^	256^[b]^	630
HCO_3_^−^	150^[d]^	515^[d]^
AcO^−^	–^[c]^	221
SO_4_^2−^	–^[e]^	–^[e]^

[a] *K*_1_ value. *K*_2_=20 m ^−1^ and 7 m ^−1^ for **4⋅(PF_6_)_2_** and **5⋅(PF_6_)_2_** respectively. [b] Chemical shift of H_β_ used. [c] Small upfield shift perturbation so binding was too weak to be quantified. [d] Anion added as tetraethylammonium salt. [e] Large downfield shifts of H_o_ gave evidence of strong sulfate binding but precipitation after one equivalent prevented calculation of *K*_Ass._

Both [3]rotaxanes exhibit a significant selectivity for nitrate over the more basic oxoanions (acetate, hydrogen carbonate and dihydrogen phosphate) and chloride. The latter is an important result considering the fact that our previous nitrate‐designed HB [2] rotaxane and [2]catenane interlocked hosts displayed comparable affinities for chloride.^22^, ^23^, ^55^ The superior nitrate recognition of this new generation of [3]rotaxanes is ascribed to the complementarity of the three‐dimensional cavity resulting from the bidentate bis‐triazolium site of the axle component and two hydrogen bond‐donating isophthalamide macrocycles. The importance of the macrocyclic wheel components was demonstrated by the significantly weaker binding of nitrate by the HB and XB axle components **1⋅(BF_4_)_2_** and **2⋅(BF_4_)_2_** in the same solvent mixture (*K*_Ass_=163 and 216 m ^−1^ respectively, Figures S23 and S24).^56^

A comparison of the association constants for each [3]rotaxane crucially reveals the XB system to demonstrate superior anion binding, for all anions, relative to the HB analogue, courtesy of strong halogen bond formation to the anionic guest. Importantly, to the best of our knowledge this is the first time an XB interlocked host system has exhibited an enhanced association for oxoanionic guests, over a comparable HB only system. In addition to the strength of the halogen bond interactions inherent to the XB [3]rotaxane, its success can also be ascribed to the stricter preference for a linear bond geometry between an XB donating iodo‐triazolium group and the anion guest, relative to that of a hydrogen bond from a proto‐triazolium motif, resulting in a more geometrically defined recognition site which may specifically aid binding of multidentate oxoanions within the cavity. The superior recognition of chloride by the XB [3]rotaxane over the HB analogue is in agreement with previously‐reported XB interlocked hosts that also exhibit improved halide binding trends.^45^, ^48^

Further structural insights on XB [3]rotaxane assembly with NO_3_^−^ and Cl^−^ anions were obtained through molecular dynamics (MD) simulations carried out at the atomic level using the GAFF^57^, ^58^ within the AMBER14 suite,^59^ using GPU cards.^60^, ^61^, ^62^ The remaining computational details are given in the SI.

Following our previous work,^50^, ^63^ the XB interactions were simulated with the inclusion, in the force field parameterisation, of an extra‐point of positive charge to represent the σ‐hole of each iodine atom of the triazolium binding units.^64^ The starting binding scenarios of **5⋅(Cl)_2_** and **5⋅NO_3_** ^**−**^ were built assembling the two macrocycles and the bis‐iodo‐triazolium axle central motif in an interlocked orthogonal binding arrangement, in agreement with the structures of analogous XB [2]rotaxane hosts.^50^, ^63^ In addition, in **5⋅(Cl)_2_**, the two chloride anions together with the two macrocycles were initially disposed in a parallel manner with each anion establishing two hydrogen bonds with a single isophthalamide binding cleft and one halogen bond with a iodo‐triazolium XB binding unit, as shown in Figure 4. Henceforth, the co‐conformation adopted by the XB [3]rotaxane **5** in this binding arrangement is called ***A***. In **5⋅NO_3_** ^**−**^ the trigonal anion was placed in a position consistent with its simultaneous recognition by the two macrocycles and the bis‐iodo‐triazolium axle central motif. These two anion [3]rotaxane arrangements, illustrated in Figure S27, were subsequently immersed in periodic cubic boxes of a solvent mixture of 1:1 CHCl_3_:CH_3_OH and their dynamic behaviours were ascertained through three MD production runs of 100 ns each.

**Figure 4:**
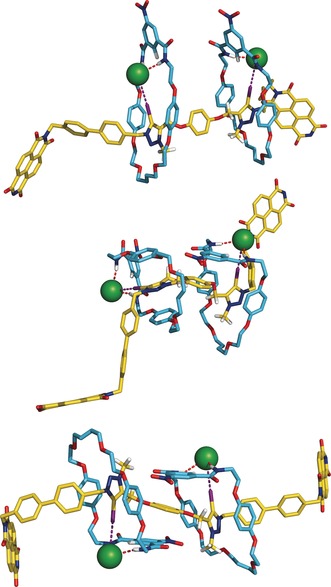
Rotational conversion of co‐conformation ***A*** (top) into co‐conformation ***C*** (bottom) of **5⋅(Cl)_2_**, passing by the intermediate co‐conformation ***B*** (middle). The bulky stoppers were removed for clarity.

During the initial nanoseconds of the three independent MD runs carried out with **5⋅(Cl)_2_**, one chloride anion coupled with its macrocycle initiates a half‐circumrotation, passing by an intermediate co‐conformation ***B*** where the macrocycles are almost perpendicular to each other. Concomitantly, the iodo‐triazolium ring halogen bonded to that chloride follows the rotating motion around the axle's main axis, while preserving the π–π stacking interactions between the electron deficient iodo‐triazolium XB binding unit and the electron rich macrocycle hydroquinone motifs, as well as the XB interactions. After this relatively short simulation period, a new binding arrangement appears, characterised by an antiparallel disposition of both macrocycles (co‐conformation ***C***) and both chlorides, and is maintained until the end of the simulation time. The two concerted rotating movements described are illustrated in Figure 4 and in Movie S1.

The conversion of co‐conformation ***A*** into ***C*** is accompanied by an increase of the distance between the chlorides by ca. 0.7 Å and a decrease of the distance between the two axle's iodine atoms of ca. 0.8 Å, as evident from the evolution of these distances throughout the simulation time plotted in Figure S28 (see SI) for the three MD runs. Throughout the MD simulation time, including the rotational events, both XB interactions are almost constantly maintained with high directional character along the three replicates with average values for the I⋅⋅⋅Cl^−^ distances and C–I⋅⋅⋅Cl^−^ angles of 3.490±0.136 Å and 173.9±3.5°, respectively. Moreover, both chloride anions are also held by the isophthalamide macrocyclic clefts via two cooperative N–H⋅⋅⋅Cl^−^ hydrogen bonds preserved during most of the MD simulation time. The N⋅⋅⋅Cl^−^ distances and N–H⋅⋅⋅Cl^−^ angles are summarised in Tables S4 and S5 for each independent MD run, along with the I⋅⋅⋅Cl^−^ distances and C–I⋅⋅⋅Cl^−^ angles. The conformational rotation observed in the three unrestrained MD simulations indicates that, in solution, **5⋅(Cl)_2_** prefers co‐conformation ***C***. Furthermore, during long simulation periods the two phenyl rings of the isophthalamide macrocyclic binding clefts (rings ***1*** and ***2***) and the hydroquinone of the bis‐iodo‐triazolium axle central motif (ring ***3***) remain almost parallel at interplanar distances consistent with the existence of stabilizing π–π stacking interactions in co‐conformation ***C***. This structural feature is particularly noticeable in two of the three MD replicates, as can be seen in Figure S28, where the variations on distances from the centroid of ***3*** to centroids ***1*** and ***2*** for 100 ns are plotted along with the ***1***‐***3***‐***2*** angle. This spatial disposition can also induce the H_o_ protons downfield chemical shift perturbations observed in the ^1^H NMR chloride titration.

Two representative snapshots of the MD simulations carried out with the assembly between the multidentate trigonal anion NO_3_^−^ and interlocked [3]rotaxane host are presented in Figure 5. In contrast with **5⋅(Cl)_2_**, the overall structure of **5⋅NO_3_** ^**−**^ oscillates between co‐conformations ***A*** (top view) and ***B*** (bottom view) along the three independent MD runs of 100 ns, with the nitrate anion tightly bonded to both macrocycles and bis‐iodo‐triazolium axle central core through HB and XB interactions, respectively. In spite of the quarter of rotation of a macrocycle relatively to each other, the assembly between the oxoanion and the three rotaxane binding entities is uninterrupted as can be seen in Movie S2. Moreover, the nitrate oxygen acceptors intermittently exchange between the isophthalamides’ N−H binding sites and C−I triazolium recognition sites, yielding slightly long average N⋅⋅⋅O (4.168±0.994 Å) and I⋅⋅⋅O (4.036±0.857 Å) distances. The swap of the nitrate's oxygen atoms between XB and HB binding sites is well depicted by the two peaks in Figures S29–S31, which show the histograms built with N⋅⋅⋅O and I⋅⋅⋅O distances observed for each MD run. However, when accounting for the oxygen atoms of NO_3_^−^ being closer to the N−H or C−I binding units (which amount to the peaks at ca. 3.1 and 2.9 Å in the histograms), the average N⋅⋅⋅O and I⋅⋅⋅O distances drop to 3.331±0.659 and 2.986±0.272 Å, typical of HB and XB interactions, respectively.

**Figure 5:**
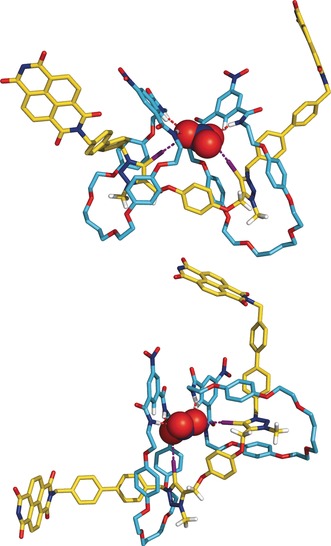
Co‐conformations ***A*** (top) and ***B*** (bottom) of **5⋅NO_3_** ^**−**^ showing the isophthalamide binding clefts almost facing each other and adopting a nearly perpendicular spatial disposition, respectively. The bulky stoppers were removed for clarity.

The different dynamic behaviours of XB complexes **5⋅(Cl)_2_** and **5⋅NO_3_** ^**−**^ are highlighted with 3D histograms built with the positions occupied by the chloride and nitrate anions within the [3]rotaxane interlocked binding pockets throughout a single MD run and shown in Figure S32. The two chloride anions display individual clouds of positions with almost hemicyclic shapes, derived from their oscillating movements around the axle, and a greater density of points mirroring the preference for the binding arrangement with **5⋅(Cl)_2_** in co‐conformation ***C***. NO_3_^−^ displays a well‐defined cloud of points constricted to the binding region defined by the isophthalamide clefts and the two iodo‐triazolium axle binding sites, with the [3]rotaxane in co‐conformation ***A***. Furthermore, the comparison between these two graphical depictions suggests that the nitrate anion is more tightly bonded to the interlocked host than the chloride anions, which is in agreement with ^1^H NMR experimental binding data.

In summary, we have synthesised the first higher‐order XB [3]rotaxane, containing a four station bis‐iodo‐triazolium‐bis‐naphthalene diimide axle and two HB‐donating macrocycle components, capable of recognising anions via a novel dynamic shuttling mechanism. Proton NMR titration experiments revealed the XB [3]rotaxane to exhibit selectivity for nitrate over more basic acetate, hydrogen carbonate and dihydrogen phosphate oxoanions, and notably chloride, and is a superior anion host in comparison to a HB [3]rotaxane analogue. The XB interlocked host achieves oxoanion recognition via both macrocycles shuttling from the peripheral NDI axle stations to the core XB iodo‐triazolium anion binding sites in a pincer‐like motion to form a unique 1:1 stoichiometric sandwich complex, as corroborated by MD simulations. The design and synthesis of dynamic higher‐order XB interlocked host systems for anion switchable and sensory applications is continuing in our laboratories.

## Supporting information

As a service to our authors and readers, this journal provides supporting information supplied by the authors. Such materials are peer reviewed and may be re‐organized for online delivery, but are not copy‐edited or typeset. Technical support issues arising from supporting information (other than missing files) should be addressed to the authors.

Supplementary

Supplementary

Supplementary
