# Supplementary material for: Selective Nitrate Recognition by a Halogen‐Bonding Four‐Station [3]Rotaxane Molecular Shuttle
Source: Angew Chem Int Ed Engl. 2016 Jul 20;55(37):11069–76. doi: 10.1002/anie.201604327 (PMC5113793; doi:10.1002/anie.201604327)
Supplement: Supplementary file 1 — Supplementary [file ANIE-55-11069-s001.pdf]

Angewandte Chemie
Eine Zeitschrift der Gesellschaft Deutscher Chemiker

Supporting Information

Selective Nitrate Recognition by a Halogen Bonding Four Station [3]Rotaxane Molecular Shuttle

*Timothy A. Barendt, Andrew Docker, Igor Marques, Vítor Félix, and Paul D. Beer**

anie_201604327_sm_miscellaneous_information.pdf
anie_201604327_sm_Movie_S1.mp4
anie_201604327_sm_Movie_S2.mp4

Contents

1) Part I: Synthesis S2
2) Part II: Additional ^{1}H NMR spectra S9
3) Part III: UV-Vis Spectroscopy S21
4) Part IV: Anion binding curves S22
5) Part V: Molecular Modelling Methods and Additional Data S28

WILEY-VCH

Part I: Synthesis

General Information

All commercial solvents and reagents were used as purchased, unless otherwise stated. Anhydrous solvents were degassed with N_2 and dried by passing them through an MBraun-800 column. Triethylamine was distilled and stored over KOH pellets. Grubbs' second generation catalyst and $Cu(MeCN)_4{\cdot}PF_6$ were stored in a desiccator with P_2O_5. TBTA was prepared following a literature procedure.[1] Water was distilled and microfiltered using a Milli-Q Millipore machine. Chromatography was undertaken using silica gel (particle size: 40-63 μm) or preparative TLC plates (20 × 20 cm, 1 cm silica thickness).
Axle and rotaxane components were fully anion exchanged using a column containing an Amberlite® ion exchange resin that had been loaded with the desired anion. Amberlite® was "loaded" by washing the resin with $NaOH_{(aq)}$ (10%), H_2O, and either $NH_4Cl_{(aq)}$ (1 M), $NH_4I_{(aq)}$ (1 M) or $NH_4PF_{6(aq)}$ (0.1 M), followed by further H_2O, and the solvent (45:45:10 $CHCl_3$:MeOH:H_2O) to be used in the anion exchange. The compound was then dissolved in 5 – 10 mL of 45:45:10 $CHCl_3$:MeOH:H_2O and passed through the column at least three times to achieve complete anion exchange. After this the solvent was removed *in vacuo*, the residue redissolved in $CHCl_3$ (5 – 10 mL) and washed with H_2O (5 – 10 mL). After drying the organic phase over anhydrous $MgSO_4$ the solvent was removed *in vacuo* to give the product.
^{1}H, ^{13}C, ^{19}F and ^{31}P NMR spectra were recorded using Bruker AVIII400 and Bruker AVIII500 spectrometers. Mass spectra were recorded on a Waters LCT Premier instrument (low resolution) or a Bruker μTOF instrument (high resolution).

The synthesis of **6,**[2] **7,**[3] **9**[4] and **3**[5] were achieved according to literature procedures.

WILEY-VCH

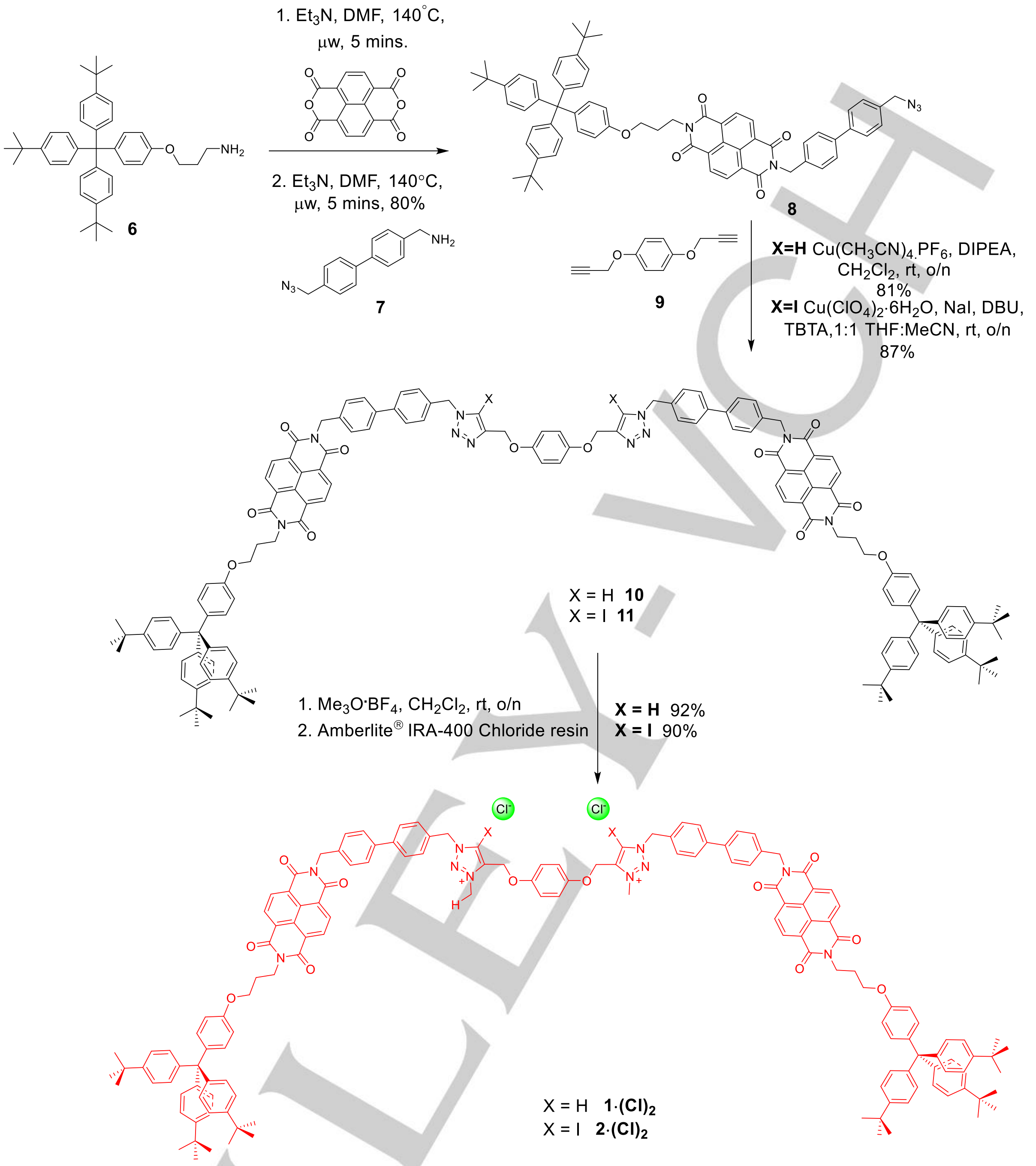

Scheme S1. Synthesis of the HB and XB multi-station axle components **1·(Cl)₂** and **2·(Cl)₂**

Synthesis of 8

Naphthalenetetracarboxylic dianhydride (52 mg, 0.194 mmol) and **6** (110 mg, 0.196 mmol) were dissolved in dry, degassed DMF (2 ml). Triethylamine (28 µl, 0.20 mmol) was then added, the vial was then sealed and heated in the microwave at 140 °C for 5 minutes. To the vial, **7** (47 mg, 0.197 mmol) and triethylamine (28µl, 0.20 mmol) were added and heated in the microwave at 140 °C for a further 5 minutes. Solvent was then removed *in vacuo* and purified by silica gel column chromatography (CH_2Cl_2) affording an orange solid (160 mg, 0.155 mmol, 80%)

^{1}H NMR (400 MHz, CHLOROFORM-*d*) δ ppm 1.30 (s, 27 H, Stopper C*H*$_3$) 2.21 - 2.31 (m, 2 H, axle C*H*$_2$) 4.10 (s, 2 H, axle C*H*$_2$) 4.38 (s, 2 H, axle C*H*$_2$) 4.41 - 4.49 (m, 2 H, axle C*H*$_2$) 5.45 (s, 2 H, axle C*H*$_2$) 6.99 - 7.09 (m, 8 H, Stopper Ar*H*) 7.23 (m, 6 H, Stopper Ar*H*) 7.37 (d, *J*=8.07 Hz, 2 H, Biphenyl Ar*H*) 7.52 - 7.60 (m, 4 H, Biphenyl Ar*H*) 7.64 (s, 4 H, Biphenyl Ar*H*) 8.70 - 8.81 (m, 4 H, NDI Ar*H*)

^{13}C NMR (101MHz, CHLOROFORM-d) δ ppm 162.9, 148.3, 144.1, 140.0, 135.8, 134.5, 132.1, 131.1, 130.7, 129.7, 128.6, 127.4, 126.6, 124.0, 112.9, 108.0, 98.4, 67.6, 66.0, 63.0, 54.5, 43.7, 38.6, 34.3, 33.2, 31.4, 28.5, 23.6

MS (MALDI): m/z calc. for $C_{68}H_{66}N_3O_5$ [M-2N+H]$^+$: 1005.50; found: 1005.35

Synthesis of 10
8 (150 mg, 0.145 mmol) and **9** (10.8 mg, 0.0581 mmol) were dissolved in dry CH_2Cl_2 (25 ml). Then TBTA (12 mg, 0.0226 mmol), $Cu(MeCN)_4{\cdot}PF_6$ (8.7 mg, 0.0233 mmol) and *N,N*-diisopropylethylamine (41 µl, 0.235 mmol) was added to the mixture and left to stir at room temperature for 2 days, monitoring by thin layer chromatography. The mixture was diluted with CH_2Cl_2 which was washed with a 0.02 M EDTA/0.1 M NH_4OH solution, the organic layer was dried over $MgSO_4$, and the solvent was removed *in vacuo*. The resultant crude was purified by silica gel column chromatography. (100 mg, 0.044 mmol, 81%)

^{1}H NMR (400 MHz, CHLOROFORM-*d*) δ ppm 1.31 (s, 54 H, Stopper C*H*$_3$) 2.20 - 2.34 (m, 4 H, Axle C*H*$_2$) 4.10 (br. s., 4 H, Axle C*H*$_2$) 4.43 (br. s., 4 H, Axle C*H*$_2$) 5.10 (s, 4 H, Axle C*H*$_2$) 5.42 (s, 4 H, Axle C*H*$_2$) 5.53 (s, 4 H, Axle C*H*$_2$) 6.64 (d, *J*=8.68 Hz, 4 H, Hydroquinone Ar*H*) 6.85 (s, 2 H, Triazole Ar*H*) 7.01 - 7.12 (m, 16 H, Stopper Ar*H*) 7.24 (d, *J*=8.31 Hz, 12 H, Stopper Ar*H*) 7.30 (d, *J*=7.95 Hz, 4 H, Biphenyl Ar*H*) 7.46 - 7.56 (m, 8 H, Biphenyl Ar*H*) 7.63 (d, *J*=8.07 Hz, 4 H, Biphenyl Ar*H*) 8.73 (q, *J*=7.58 Hz, 8 H, NDI Ar*H*)

^{13}C NMR (101 MHz, CHLOROFORM-*d*) δ ppm 27.97, 31.41, 34.31, 38.67, 43.71, 53.88, 62.71, 63.06, 65.99, 112.95, 115.84, 122.60, 124.06, 126.53, 126.72, 127.26, 127.72, 128.56, 129.74, 130.72, 131.02, 131.19, 132.19, 133.55, 136.08, 139.71,141.21, 144.14, 144.78, 148.32, 152.76, 156.52, 162.85

MS (ESI): m/z calc. for $C_{148}H_{142}N_{10}NaO_{12}$ [M + Na + H + H]$^{3+}$:758.36 found; 759.31

Synthesis of 11
8 (100 mg, 0.0969 mmol) was dissolved in dry degassed THF (2 ml), NaI (46.1 mg, 0.307 mmol) was added followed by $Cu(ClO_4).6H_2O$, which was left stirring for 5 minutes. TBTA (4 mg, 0.0077 mmol) and DBU (11.7 mg, 0.0768 mmol) were added followed by **9** (7.07 mg, 0.038 mmol). The mixture was left to stir at room temperature for 2 days, avoiding exposure to light. The mixture was diluted with CH_2Cl_2 then washed with a 0.02 M EDTA/0.1 M NH_4OH solution, the organic layer was dried over $MgSO_4$, and the solvent was removed *in vacuo*. The resultant solid was purified by silica gel column chromatography (99:1 CH_2Cl_2:MeOH), (83 mg, 0.033 mmol, 87%).

^{1}H NMR (400 MHz, CHLOROFORM-*d*) δ ppm 1.21 - 1.38 (m, 54 H, Stopper C*H*$_3$) 2.26 (d, *J*=7.34 Hz, 4 H, axle C*H*$_2$) 4.10 (s, 4 H, axle C*H*$_2$) 4.43 (s, 4 H, axle C*H*$_2$) 5.05 (s, 4

WILEY-VCH

H, axle C*H*$_2$) 5.42 (s, 4 H, axle C*H*$_2$) 5.60 (s, 4 H, axle C*H*$_2$) 6.64 (d, *J*=8.80 Hz, 4 H, Hydroquinone Ar*H*) 6.94 (s, 4 H, Stopper Ar*H*) 6.98 - 7.10 (m, 16 H, Stopper Ar*H*) 7.23 (d, *J*=8.44 Hz, 12 H, Stopper Ar*H*) 7.31 (d, *J*=8.19 Hz, 4 H, Biphenyl Ar*H*) 7.51 (d, *J*=7.82 Hz, 8 H, Biphenyl Ar*H*) 7.62 (d, *J*=8.19 Hz, 4 H, Biphenyl Ar*H*) 8.69 - 8.79 (m, 8 H, NDI Ar*H*)

^{13}C NMR (101MHz, CHLOROFORM-d) δ ppm 162.8, 156.5, 152.9, 148.1, 144.1, 140.1, 135.9, 133.1, 132.1, 131.1, 130.7, 129.7, 128.3, 127.5, 127.2, 126.7, 126.5, 124.0, 116.3, 112.9, 98.4, 80.5, 68.4, 67.4, 65.9, 63.0, 62.5, 53.9, 53.4, 43.7, 38.6, 34.3, 33.2, 31.4, 27.8, 23.3

MS (ESI): m/z calc. $C_{148}H_{138}I_2N_{10}O_{12}Na$ [M + Na]$^+$: 2525.85; found: 2525.09

Synthesis of 1·(Cl)$_2$
10 (100 mg, 0.0444 mmol) was dissolved in CH_2Cl_2 (40 ml), $Me_3O{\cdot}BF_4$ (14.5 mg, 0.0981 mmol) was also added and the mixture was left stirring overnight at room temperature. The reaction was quenched with three drops of MeOH and the solvent was removed *in vacuo*. The resultant solid was purified by preparative silica TLC (CH_2Cl_2:MeOH 97:3) and isolated as **1·(BF$_4$)$_2$**. Subsequent anion exchange was achieved to yield **1·(Cl)$_2$**, by dissolving **1·(BF$_4$)$_2$** in (45:45:10 $CHCl_3$:MeOH:H_2O) and passing the solution three times through a column of Amberlite® resin loaded with chloride. (96 mg, 0.041 mmol, 92%).

^{1}H NMR (400 MHz, CHLOROFORM-*d*) δ ppm 1.30 (s, 54 H, Stopper C*H$_3$*) 2.16 - 2.27 (m, 4 H, Axle C*H$_2$*) 4.03 - 4.12 (m, 4 H, Axle C*H$_2$*) 4.36 (br. s., 10 H, Axle C*H*$_2$, Triazolium C*H$_3$*) 5.35 (s, 4 H, Axle C*H$_2$*) 5.46 - 5.54 (m, 4H, Axle C*H$_2$*) 5.88 - 5.97 (m, 4 H, Axle C*H$_2$*) 6.64 (d, *J*=8.93 Hz, 4 H, Hydroqinone Ar*H*) 6.94 - 6.99 (m, 2 H, Triazolium Ar*H*) 6.99 - 7.09 (m, 18 H, Stopper Ar*H*) 7.22 (d, *J*=8.56 Hz, 12 H, Stopper Ar*H*) 7.41 - 7.51 (m, 8 H, Biphenyl, Ar*H*) 7.57 (d, *J*=8.31 Hz, 8 H, Biphenyl Ar*H*) 8.69 (d, *J*=6.85 Hz, 8 H, NDI Ar*H*)

^{13}C NMR (101 MHz, CHLOROFORM-*d*) δ ppm 31.37, 34.27, 63.02, 65.31, 66.45, 112.92, 124.01 126.04, 126.61, 126.97, 127.17, 127.28, 128.06, 129.67, 130.23, 131.37, 132.16, 138.83, 141.26, 144.12, 148.27, 156.50, 162.72
MS (ESI): m/z calc. for $C_{150}H_{146}N_{10}O_{12}$ [M - 2BF$_4$]$^{2+}$ = 1140.05721 found 1140.05224

Synthesis of 2·(Cl)$_2$
11 (83 mg, 0.037 mmol) was dissolved in CH_2Cl_2 (33 ml), $Me_3O{\cdot}BF_4$ (10.8 mg, 0.07320 mmol) was also added and the mixture was left stirring overnight at room temperature. The reaction was quenched with three drops of MeOH and the solvent was removed *in vacuo*. The resultant solid was purified by preparative silica TLC (CH_2Cl_2:MeOH 97:3) and isolated as **2·(BF$_4$)$_2$**. Subsequent anion exchange was achieved to yield **2·(Cl)$_2$**, by dissolving **2·(BF$_4$)$_2$** in (45:45:10 $CHCl_3$:MeOH:H_2O) and passing the solution three times through a column of Amberlite® resin loaded with chloride. (86 mg, 0.033 mmol, 90%).

^{1}H NMR (400 MHz, CHLOROFORM-*d*) δ ppm 1.30 (s, 54 H, Stopper C*H$_3$*) 2.16 - 2.29 (m, 4 H, Axle C*H$_2$*) 4.04 - 4.12 (m, 4 H, Axle C*H$_2$*) 4.37 - 4.50 (m, 10 H, Axle C*H$_2$* and Triazolium C*H$_3$*) 5.32 - 5.38 (m, 4 H, Axle C*H$_2$*) 5.42 - 5.48 (m, 4 H, Axle C*H$_2$*) 5.58 - 5.63 (m, 4 H, Axle C*H*$_2$) 6.61 - 6.69 (m, 4 H, Hydroquinone Ar*H*) 6.91 - 6.96 (m, 4 H,

WILEY-VCH

Stopper Ar*H*) 7.06 (d, *J*=8.68 Hz, 14 H, Stopper Ar*H*) 7.21 (s, 14 H, Biphenyl and Stopper Ar*H*) 7.37 - 7.62 (m, 14 H, Biphenyl and Stopper Ar*H*) 8.60 - 8.76 (m, 8 H, NDI Ar*H*)

^{13}C NMR (101 MHz, CHLOROFORM-*d*) δ ppm 31.35, 34.26, 112.90, 124.00, 126.61, 127.14,127.56, 129.07, 129.75, 130.68, 130.79, 132.15, 139.17, 144.09, 148.27, 155.98, 162.73

MS (ESI): *m/z* calc. for $C_{150}H_{144}I_2N_{16}O_{12}$ [M - 2BF$_4$]$^{2+}$ = 1265.95386 found 1265.95012

WILEY-VCH

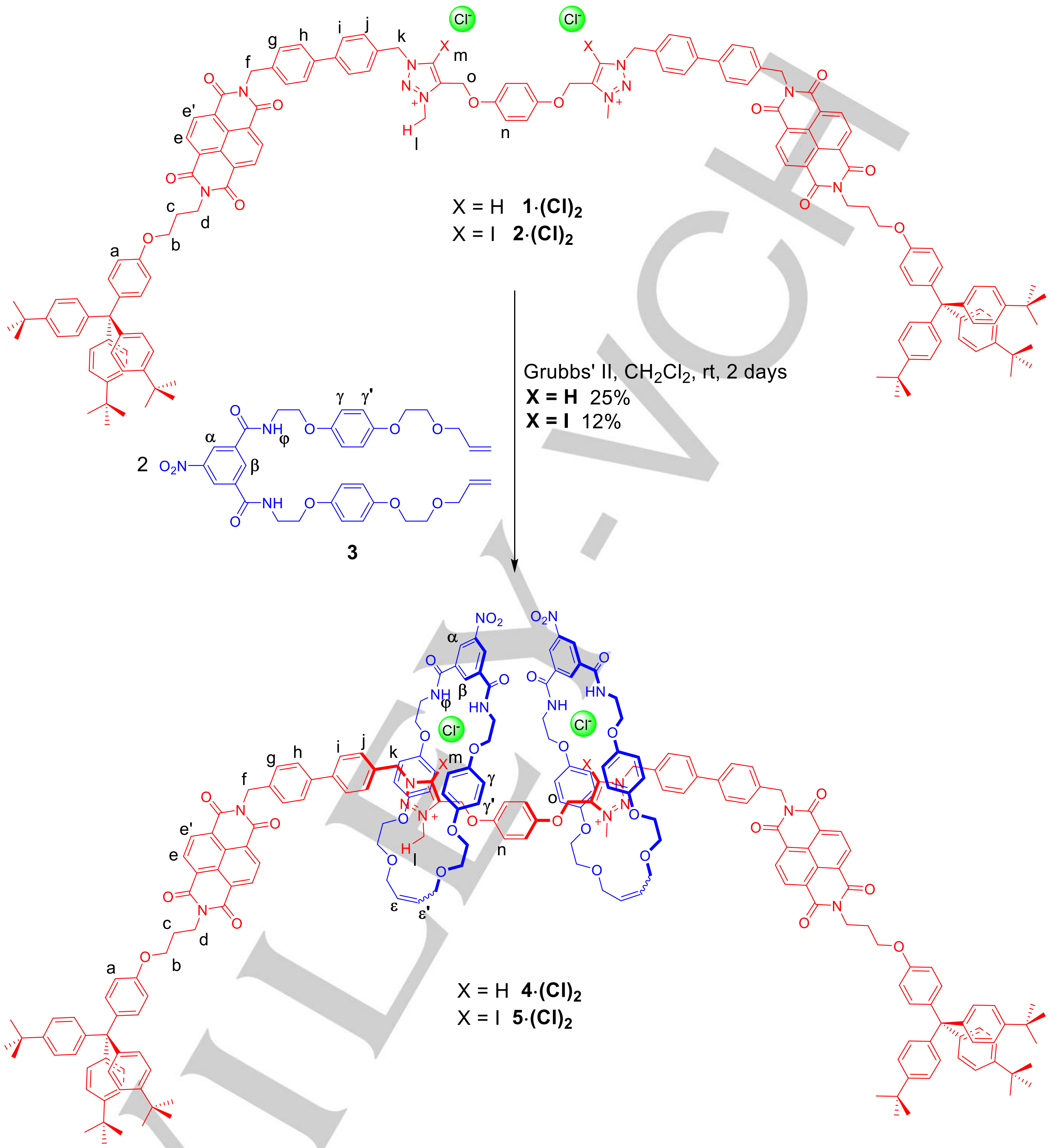

Scheme S2. Synthesis of the HB and XB multi-station [3]rotaxanes **4·(Cl)₂** and **5·(Cl)₂**, indicating the predicted co-conformation of the molecules as their chloride salts

Synthesis of 4·(Cl)$_2$

1·(Cl)$_2$ (21 mg, 0.00892 mmol) and **3** (16 mg, 0.0246 mmol) were dissolved in dry CH_2Cl_2 (5 ml), the mixture was left to stir for 30 minutes. Grubbs' second generation catalyst (1.6 mg, 10 wt. %) was added and the solution stirred at room temperature,

monitoring by TLC and ESI mass spectrometry throughout. After two days a further addition of Grubbs' second generation catalyst (1.6 mg, 10 wt. %) was made and after three days the solvent was removed *in vacuo*. The crude was purified by iterative preparative silica TLC using the solvent mixtures (EtOAc:MeOH 95:5) and (CH_2Cl_2:MeOH 95:5) respectively. (9 mg, 2.23 µmol, 25%)

^{1}H NMR (400 MHz, CHLOROFORM-*d*) δ ppm 1.30 (s, 54 H, Stopper C*H₃*) 2.41 (s, 4 H, C*H₂*) 3.70 - 4.15 (m, 54 H, C*H₂* and Triazolium C*H₃*) 4.37 - 4.48 (m, 4 H, C*H₂*) 5.44 (s, 4 H, C*H₂*) 5.59 - 5.66 (m, 4 H, C*H₂*) 5.87 (br. s., 4 H, C*H₂*) 6.19 - 6.46 (m, 16 H, Hydroquinone Ar*H*) 6.65 (d, *J*=8.80 Hz, 4 H, Hydroquinone Ar*H*) 6.94 - 7.26 (m, 32 H, Stopper Ar*H*) 7.46 - 8.00 (m, 16 H, Biphenyl Ar*H*) 8.61 - 8.85 (m, 12 H, NDI and Macrocycle Ar*H*) 9.29 - 9.48 (m, 2 H, Axle Triazolium Ar*H*) 9.79 - 10.12 (m, 2 H, Macrocycle Isophthalamide Ar*H*)

^{13}C NMR (101 MHz, CHLOROFORM-*d*) δ ppm 17.66, 20.81, 31.38, 34.30, 62.76, 65.78, 66.06, 67.79 - 69.03, 70.75, 112.93, 114.43, 115.02, 115.44, 124.03, 125.84, 126.36, 126.77, 127.35, 127.65 128.91, 129.84, 130.14, 130.71, 130.92, 131.12, 132.18, 134.29, 136.08, 137.17, 139.29, 140.53, 144.12, 148.31, 151.99, 153.08, 156.72, 164.21

MS (ESI): *m/z* calc. for $C_{214}H_{216}N_{16}O_{32}$ $[M - 2Cl]^{2+}$ = 1761.79414 found 1761.79114

For 4·$(NO_3)_2$: MS (ESI): *m/z* calc. for $C_{214}H_{216}KN_{18}O_{38}^+$ $[M + K]^+$ = 3686.52 found 3686.54

Synthesis of 5·$(Cl)_2$

2·$(Cl)_2$ (21.3 mg, 0.00821 mmol) and **3** (16 mg, 0.0246 mmol) were dissolved in dry CH_2Cl_2 (5 ml) and were left to stir for 30 minutes. Grubbs' second generation catalyst (1.6 mg, 10 wt. %) was added and the solution stirred at room temperature, monitoring by TLC and ESI mass spectrometry throughout. After two days a further addition of Grubbs' second generation catalyst (1.6 mg, 10 wt. %) was made and after three days the solvent was removed *in vacuo*. The crude was purified by iterative preparative silica TLC (EtOAC:MeOH 95:5) then (CH_2Cl_2:MeOH 95:5). (3 mg, 0.9 µmol 12%)

^{1}H NMR (400 MHz, CHLOROFORM-*d*) δ ppm 1.30 (s, 54 H, Stopper C*H₃*) 2.22 - 2.42 (m, 6 H, C*H₂*) 3.78 (br. s., 16 H, C*H₂*) 4.02 - 4.20 (m, 26 H, C*H₂*) 4.28 (s, 6 H, C*H₂*) 4.37 - 4.48 (m, 6 H, Triazolium C*H₃*) 5.09 (br. s., 4 H, C*H₂*) 5.45 (s, 4 H, C*H₂*) 5.98 (br. s., 2 H, Macrocycle alkene C*H*) 6.24 (d, *J*=8.68 Hz, 4 H, Hydroquinone Ar*H*) 6.36 (d, *J*=8.56 Hz, 4 H, Hydroquinone Ar*H*) 6.57 - 6.70 (m, 4 H, Hydroquinone Ar*H*) 6.99 - 7.11 (m, 16 H, Stopper and Biphenyl Ar*H*) 7.23 (d, *J*=8.44 Hz, 12 H, Stopper and Biphenyl Ar*H*) 7.40 - 7.47 (m, 4 H, Stopper Ar*H*) 7.59 (d, *J*=5.14 Hz, 8 H, Stopper Ar*H*) 7.66 (s, 8 H, Stopper Ar*H*) 8.76 (d, *J*=7.82 Hz, 8 H, NDI Ar*H*) 8.94 (s, 6 H, Macrocycle Isophthalamide Ar*H*) 9.46 - 9.55 (m, 2 H, Macrocycle Amide NH)

^{13}C NMR (101 MHz, CHLOROFORM-*d*) δ ppm 18.01 31.38 34.30 62.94 65.88 67.88 69.16 70.82 112.93 114.43 115.01 115.44 124.03 126.44 126.77 127.35 128.91 129.84 130.14 130.29 132.18 134.80 136.42 137.02 139.13 140.27 144.12 148.31 147.62 151.87 155.88 156.89 162.82 163.36

MS (ESI): *m/z* calc. for $C_{214}H_{214}I_2N_{16}O_{32}$ $[M - 2Cl]^{2+}$ = 1887.68778 found 1887.68682

Part II: Additional ^{1}H NMR spectra

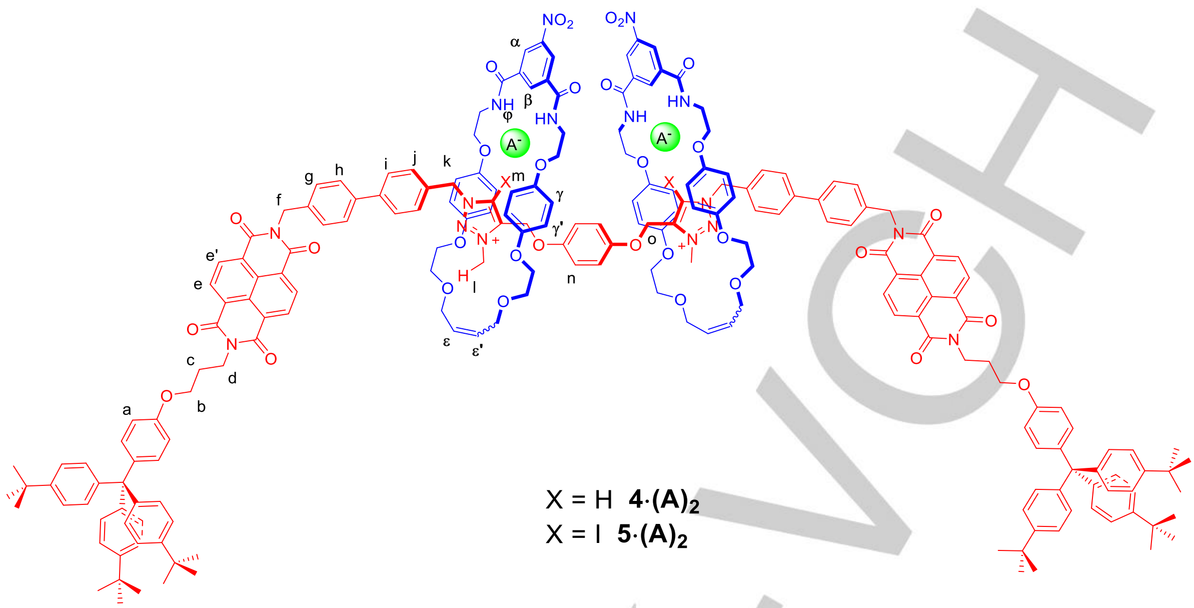

X = H **4·(A)$_2$**
X = I **5·(A)$_2$**

2D ROESY NMR of HB [3]rotaxane 4·(Cl)$_2$

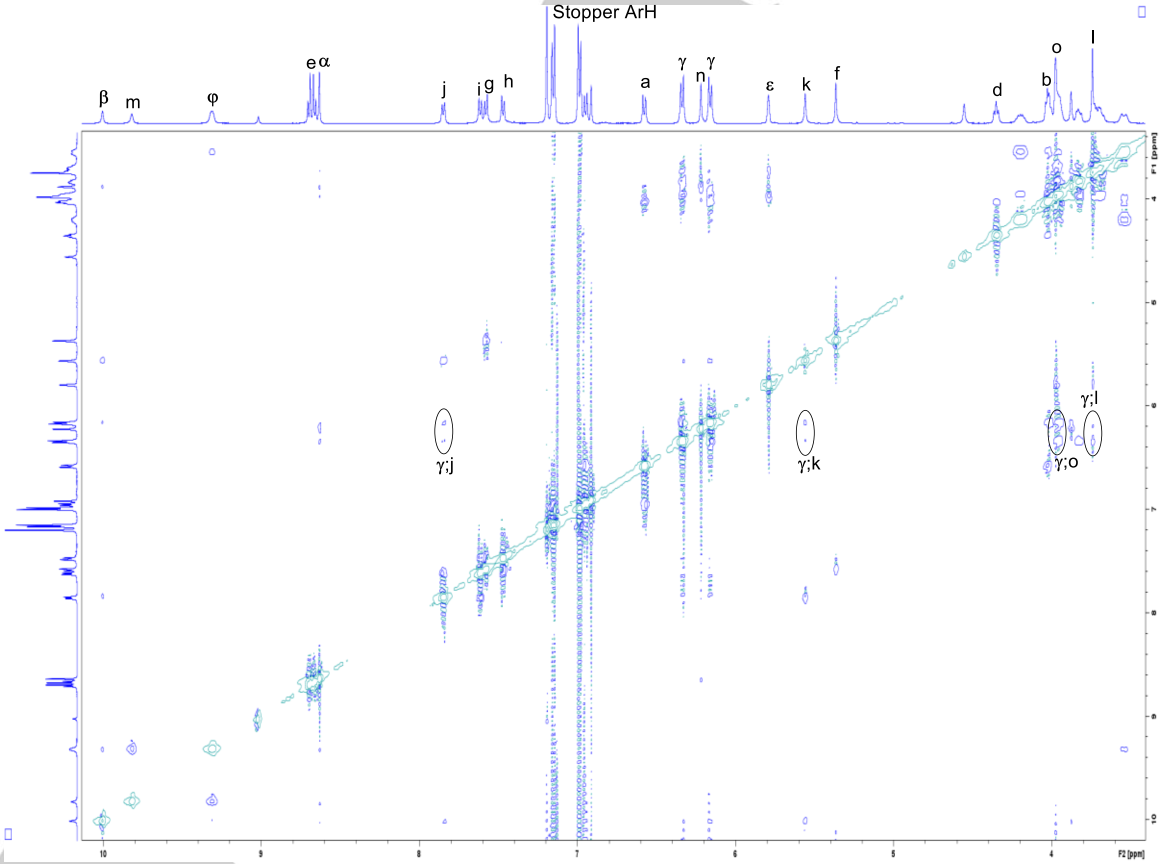

Figure S1. Truncated ^{1}H-^{1}H ROESY NMR spectrum of HB rotaxane **4·(Cl)$_2$** with highlighted coupling interactions indicating predominant macrocycle occupancy of the triazolium stations ($CDCl_3$, 298 K, 500 MHz).

2D ROESY NMR of XB [3]rotaxane 5·(Cl)$_2$

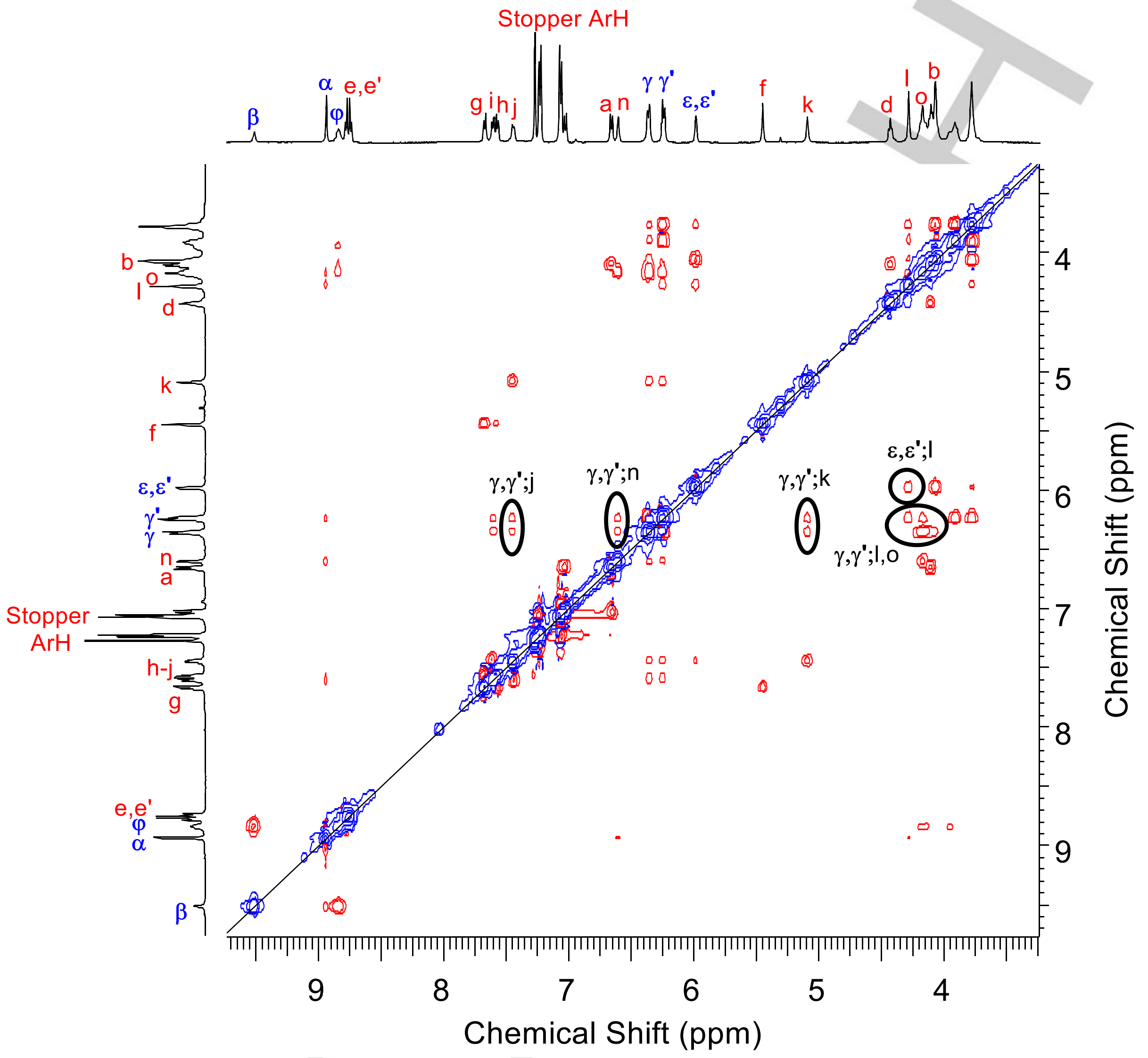

Figure S2. Truncated ^{1}H-^{1}H ROESY NMR spectrum of XB rotaxane **5·(Cl)$_2$** with highlighted coupling interactions indicating predominant macrocycle occupancy of the triazolium stations ($CDCl_3$, 298 K, 500 MHz).

HB [3]rotaxane *vs*. HB axle NMR stacks: coordinating anion salts

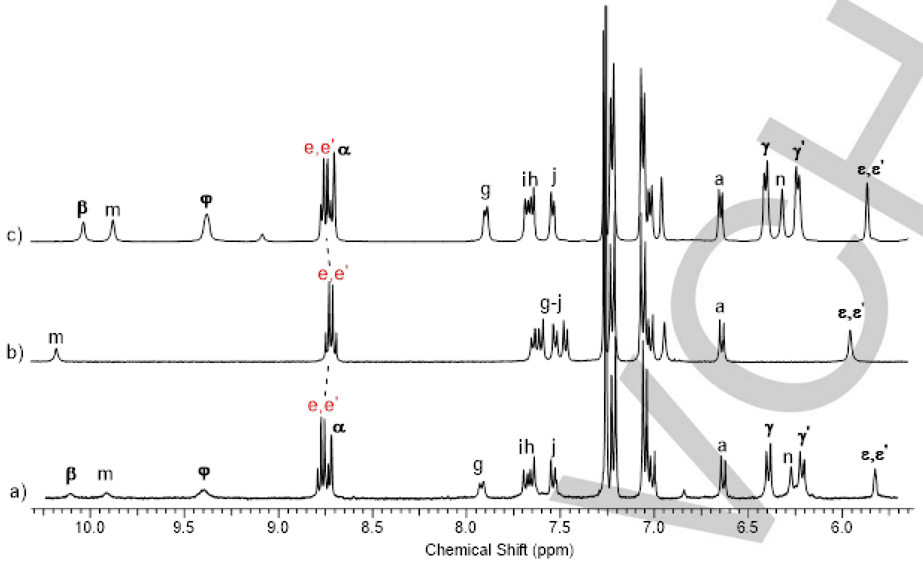

Figure S3. Truncated ^{1}H NMR spectra of a) [3]rotaxane **4·(PF$_6$)·(NO$_3$)**, b) axle **1·(Cl)$_2$** and c) [3]rotaxane **4·(Cl)$_2$**. In the presence of coordinating anions the signals for $H_{e,e'}$ are unperturbed in the [3]rotaxanes relative to the axle component revealing the macrocycle components undergo negligible interactions with the NDI stations (CDCl$_3$, 298 K, 500 MHz).

WILEY-VCH

XB [3]rotaxane *vs*. XB axle stacks: coordinating anion salts

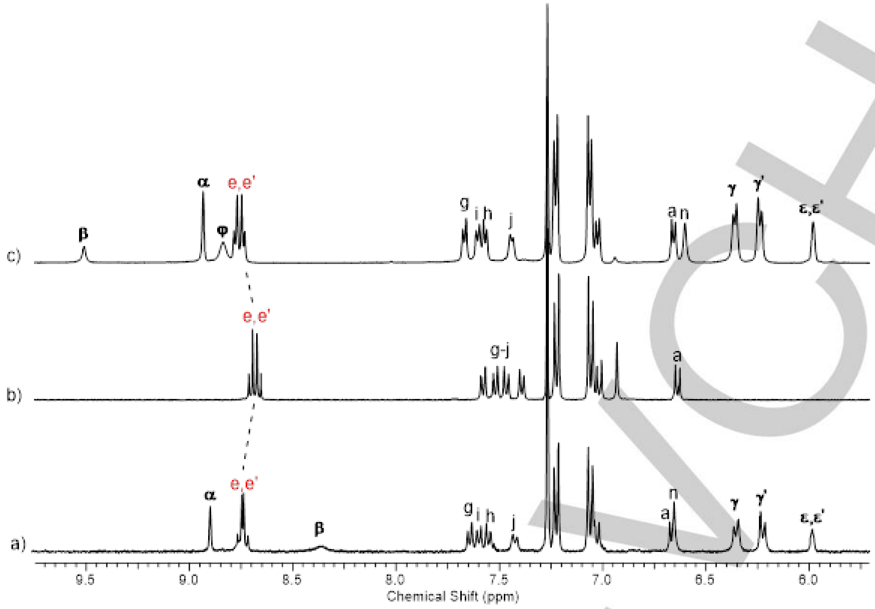

Figure S4. Truncated ^{1}H NMR spectra of a) [3]rotaxane **5·(PF$_6$)·(NO$_3$)**, b) axle **2·(Cl)$_2$** and c) [3]rotaxane **5·(Cl)$_2$**. In the presence of coordinating anions the signals for $H_{e,e'}$ are perturbed downfield in the [3]rotaxanes relative to the axle component revealing the macrocycle components undergo negligible interactions with the NDI stations (CDCl$_3$, 298 K, 500 MHz).

WILEY-VCH

HB [3]rotaxane NMR stacks: coordinating and non-coordinating anion salts

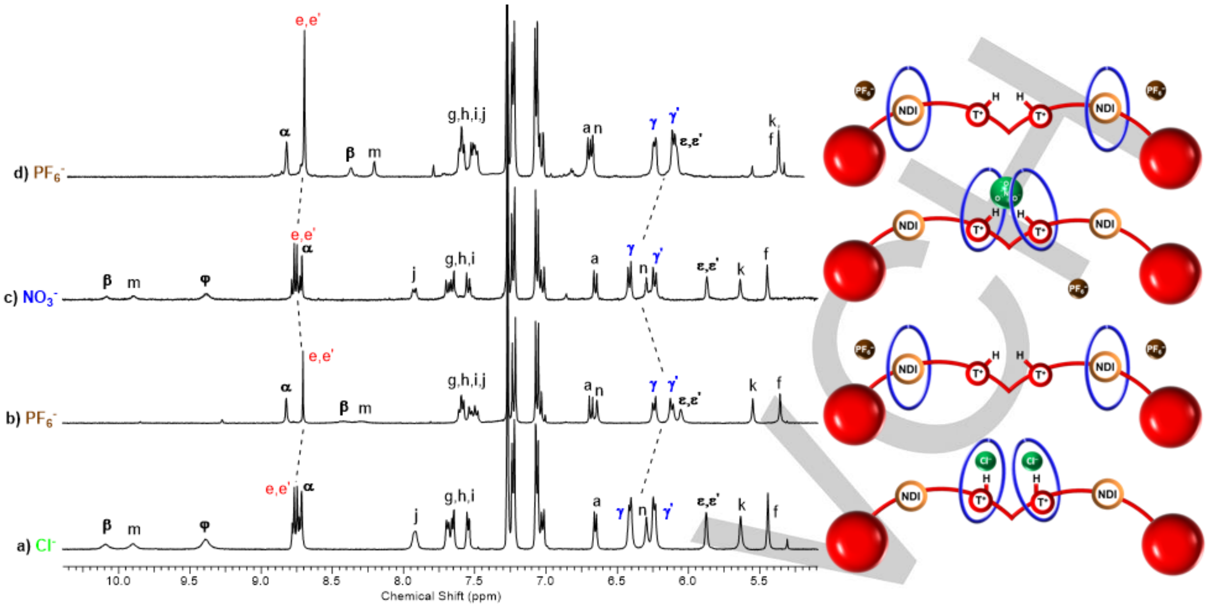

Figure S5. Truncated ^{1}H NMR spectra of HB [3]rotaxanes a) **4·(Cl)$_2$**, b) **4·(PF$_6$)$_2$**, c) **4·(NO$_3$)·(PF$_6$)** and d) **4·(PF$_6$)$_2$** demonstrating the full operational cycle of the shuttle ($CDCl_3$, 298 K, 400 MHz). The sample in b) was prepared from a) using Amberlite® anion exchange resin. The sample in c) was prepared from b) by the addition of one equivalent of TBA$(NO_3)_{(s)}$. The sample in d) was prepared from c) using an excess of $NaPF_{6(s)}$.

WILEY-VCH

2D ROESY NMR of HB [3]rotaxane 4·(PF6)2

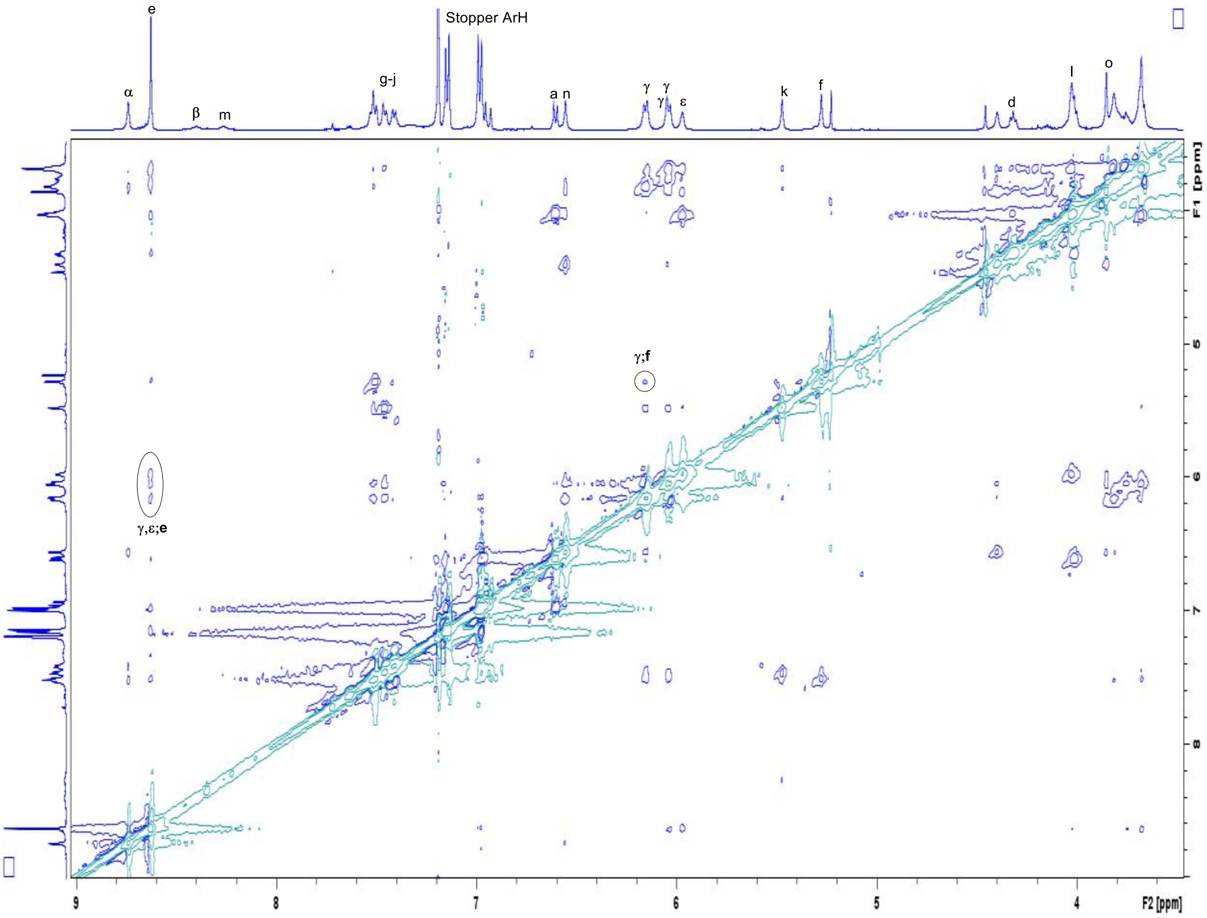

Figure S6. Truncated ^{1}H-^{1}H ROESY NMR spectrum of rotaxane **4·(PF$_6$)$_2$** with highlighted coupling interactions indicating a significant macrocycle occupancy of the NDI stations ($CDCl_3$, 298 K, 500 MHz).

WILEY

2D ROESY NMR of XB [3]rotaxane 5·(PF$_6$)$_2$

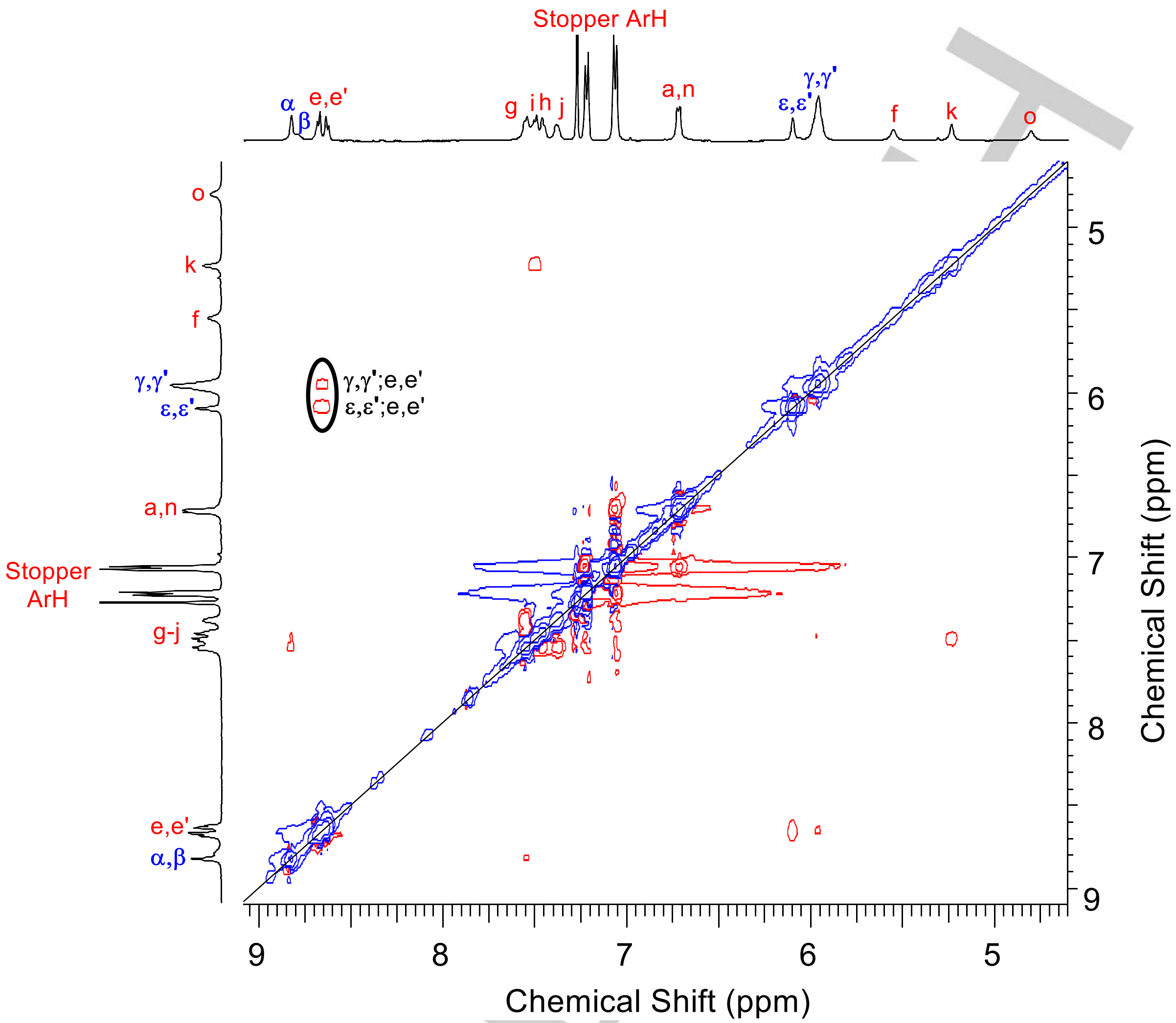

Figure S7. Truncated ^{1}H-^{1}H ROESY NMR spectrum of rotaxane **5·(PF$_6$)$_2$** with highlighted coupling interactions indicating a significant macrocycle occupancy of the NDI stations (CDCl$_3$, 298 K, 500 MHz).

WILEY

HB [3]rotaxane *vs*. HB axle NMR stacks: non-coordinating anion salt

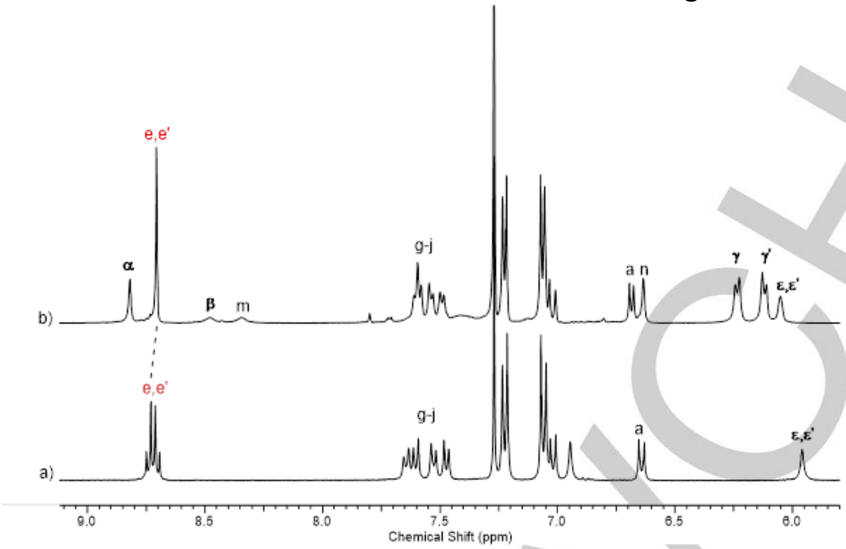

Figure S8. Truncated ^{1}H NMR spectra of a) axle **1·(PF$_6$)$_2$** and b) [3]rotaxane **4·(PF$_6$)$_2$.** In the absence of coordinating anions the signals for $H_{e,e'}$ are perturbed upfield in the [3]rotaxane relative to the axle component revealing the macrocycle components interact significantly with the NDI stations (CDCl$_3$, 298 K, 500 MHz).

WILEY-VCH

XB [3]rotaxane *vs*. XB axle NMR stacks: non-coordinating anion salt

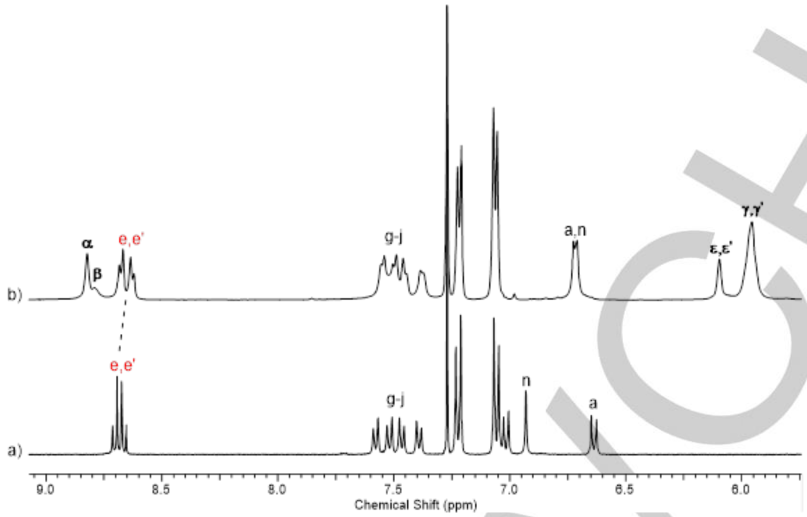

Figure S9. Truncated ^{1}H NMR spectra of a) axle **2·(PF$_6$)$_2$** and b) [3]rotaxane **5·(PF$_6$)$_2$**. In the absence of coordinating anions the signals for $H_{e,e'}$ are perturbed upfield in the [3]rotaxane relative to the axle component revealing the macrocycle components interact significantly with the NDI stations ($CDCl_3$, 298 K, 500 MHz).

WILEY-VCH

2D ROESY NMR of HB [3]rotaxane 4·(PF6)·(NO3)

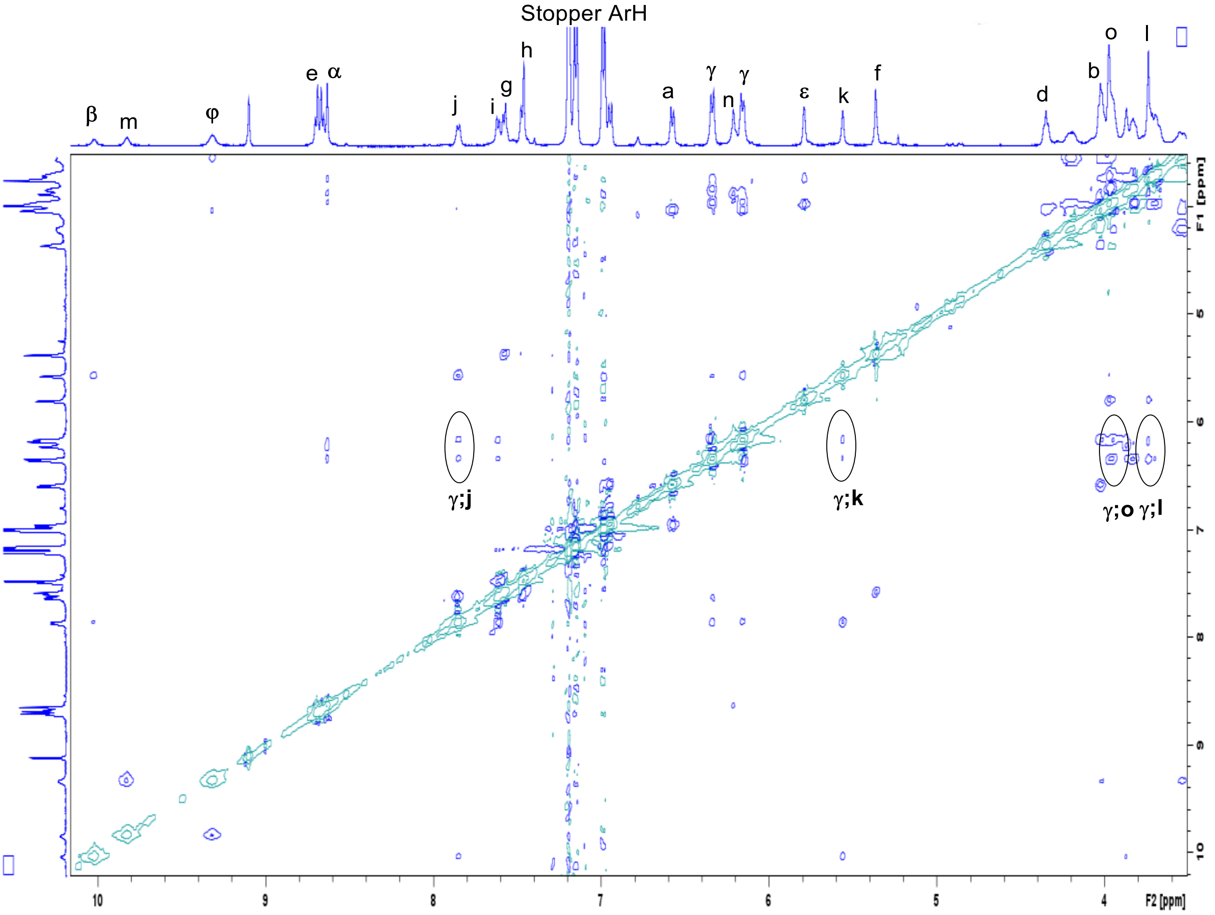

Figure S10. Truncated ^1H-^1H ROESY NMR spectrum of rotaxane **4·(PF6)·(NO3)** with highlighted coupling interactions indicating predominant macrocycle occupancy of the triazolium stations ($CDCl_3$, 298 K, 500 MHz).

WILEY

2D ROESY NMR of XB [3]rotaxane 5·(PF$_6$)·(NO$_3$)

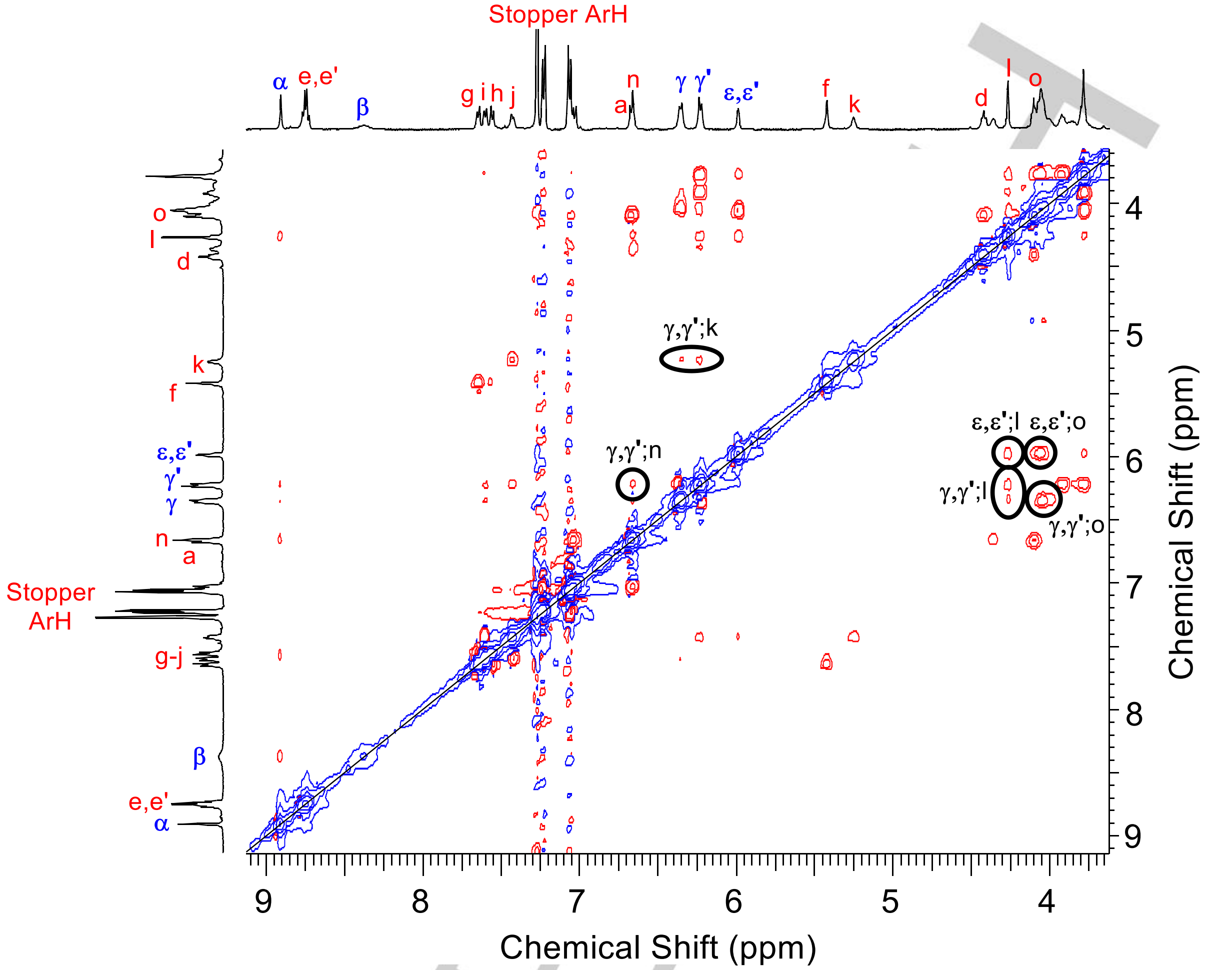

Figure S11. Truncated ^{1}H-^{1}H ROESY NMR spectrum of XB [3]rotaxane **5·(PF$_6$)·(NO$_3$)** with highlighted coupling interactions indicating predominant macrocycle occupancy of the triazolium stations ($CDCl_3$, 298 K, 500 MHz).

WILEY

XB [3]rotaxane NMR stacks: coordinating and non-coordinating anion salts

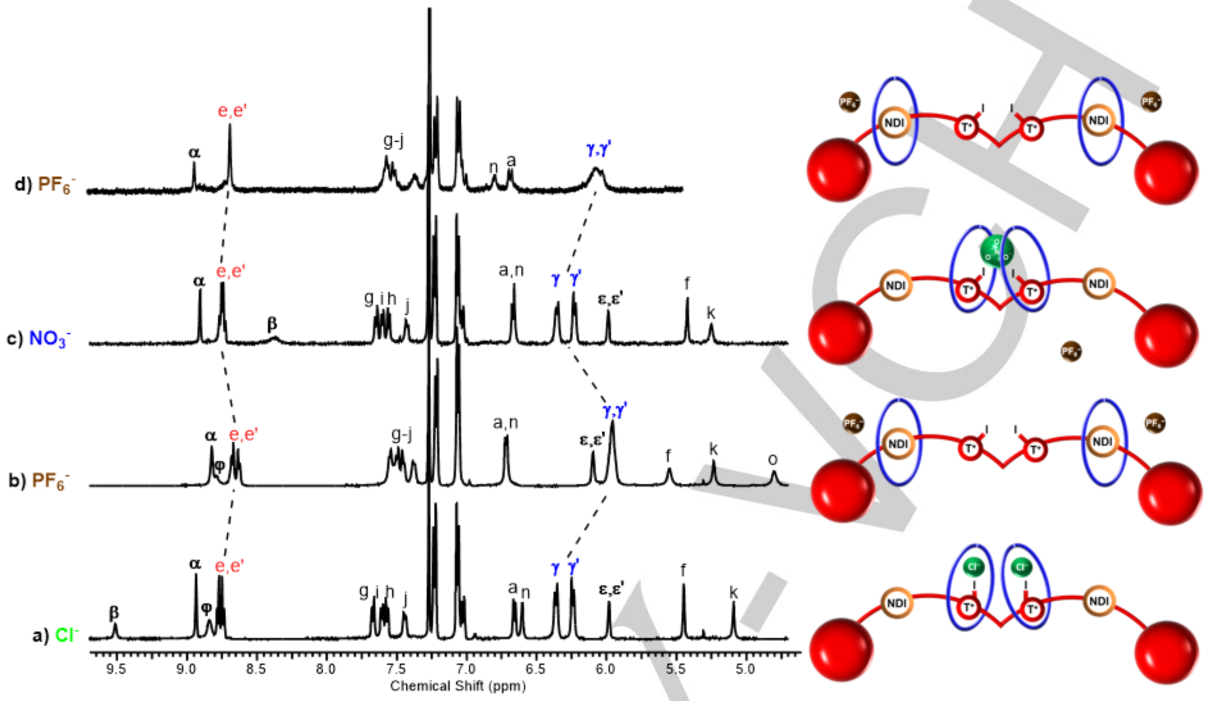

Figure S12. Truncated ^{1}H NMR spectra of XB [3]rotaxanes a) **5·(Cl)$_2$**, b) **5·(PF$_6$)$_2$**, c) **5·(NO$_3$)·(PF$_6$)** and d) **5·(PF$_6$)$_2$** demonstrating the full operational cycle of the shuttle ($CDCl_3$, 298 K, 400 MHz). The sample in b) was prepared from a) using Amberlite® anion exchange resin. The sample in c) was prepared from b) by the addition of one equivalent of TBA(NO_3)$_{(s)}$. The sample in d) was prepared from c) using an excess of NaPF$_{6(s)}$.

WILEY-VCH

Part III: UV-Vis Spectroscopy Data

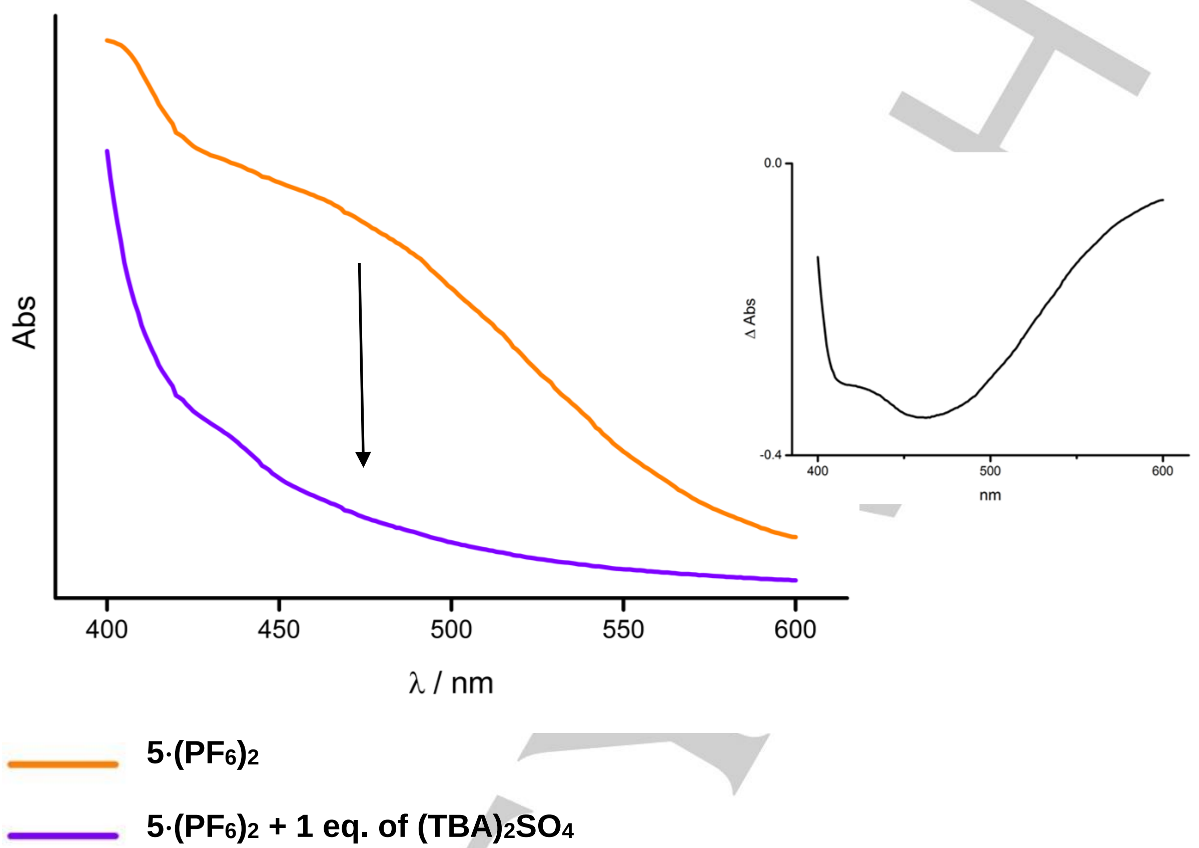

Figure S13. UV-Vis spectra showing the effect of the addition of one equivalent of tetrabutylammonium sulfate into a solution of rotaxane **5·(PF$_6$)$_2$** in $CHCl_3$ (1×10^{-3} mol L^{-1}). The addition of sulfate leads to a decrease in intensity of the charge–transfer band.

Part IV: Anion binding curves

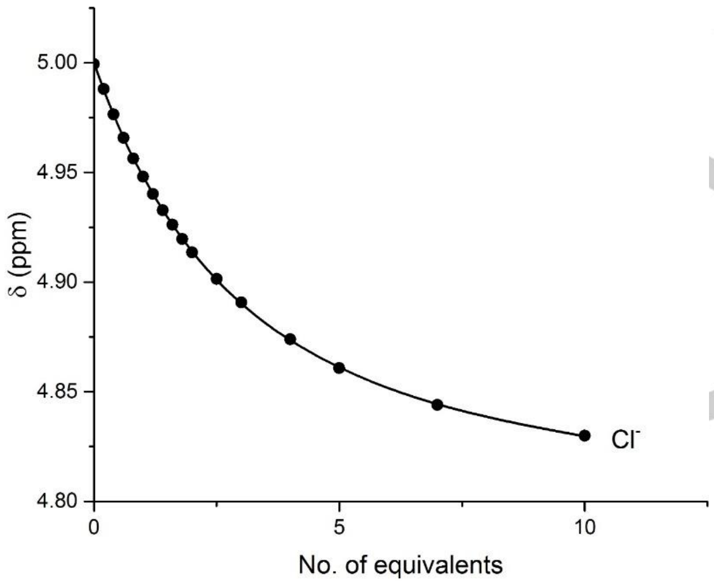

Figure S14. Titration curve for HB [3]rotaxane **4·(PF$_6$)$_2$** with chloride. Curve obtained by monitoring the proton H$_o$ by ^{1}H NMR spectroscopy in (1:1 CDCl$_3$/CD$_3$OD, 298 K, 500 MHz). Solid points represent experimental data; continuous line represents theoretical binding isotherm.

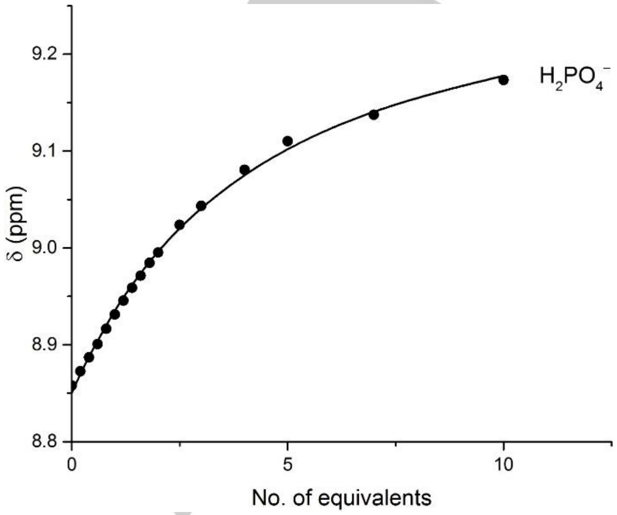

Figure S15. Titration curve for HB [3]rotaxane **4·(PF$_6$)$_2$** with dihydrogen phosphate. Curve obtained by monitoring the proton H$_β$ by ^{1}H NMR spectroscopy in (1:1 CDCl$_3$/CD$_3$OD, 298 K, 500 MHz). Solid points represent experimental data; continuous line represents theoretical binding isotherm.

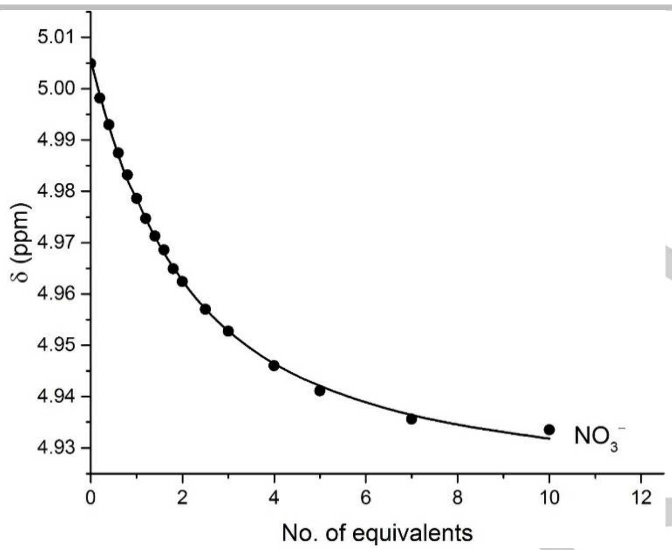

Figure S16. Titration curve for HB [3]rotaxane **4·(PF$_6$)$_2$** with nitrate. Curve obtained by monitoring the proton H$_o$ by ^{1}H NMR spectroscopy in (1:1 $CDCl_3$/CD_3OD, 298 K, 500 MHz). Solid points represent experimental data; continuous line represents theoretical binding isotherms.

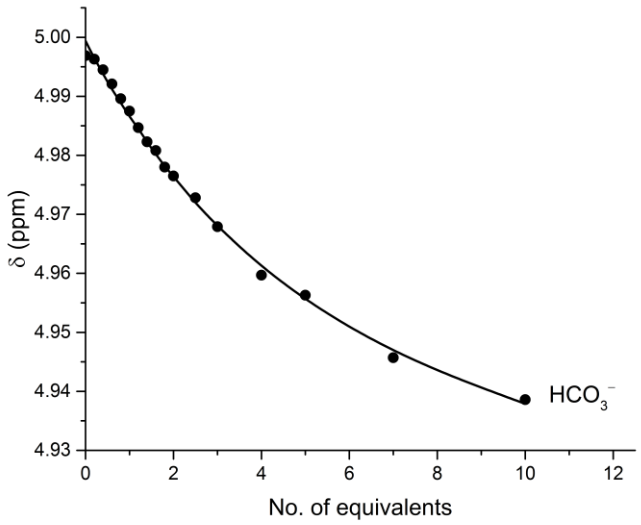

Figure S17. Titration curve for HB [3]rotaxane **4·(PF$_6$)$_2$** with hydrogen carbonate. Curve obtained by monitoring the proton H$_o$ by ^{1}H NMR spectroscopy in (1:1 $CDCl_3$/CD_3OD, 298 K, 500 MHz). Solid points represent experimental data; continuous line represents theoretical binding isotherms.

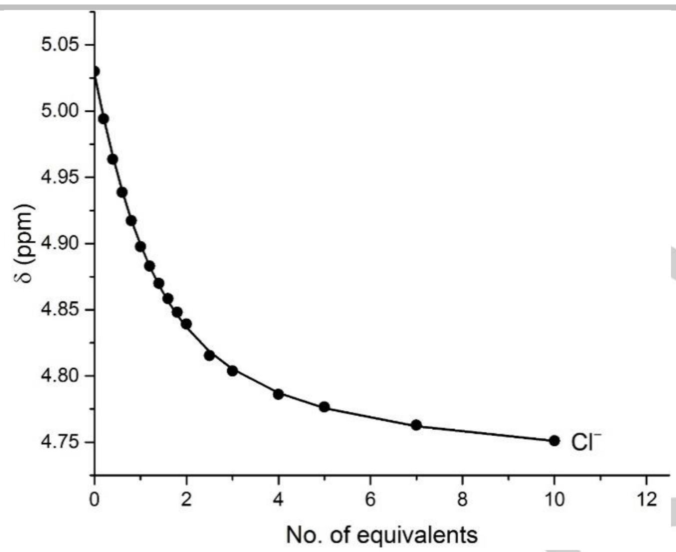

Figure S18. Titration curve for XB [3]rotaxane **5·(PF$_6$)$_2$** with chloride. Curve obtained by monitoring the proton H$_o$ by ^{1}H NMR spectroscopy in (1:1 $CDCl_3$/CD_3OD, 298 K, 500 MHz). Solid points represent experimental data; continuous line represents theoretical binding isotherm.

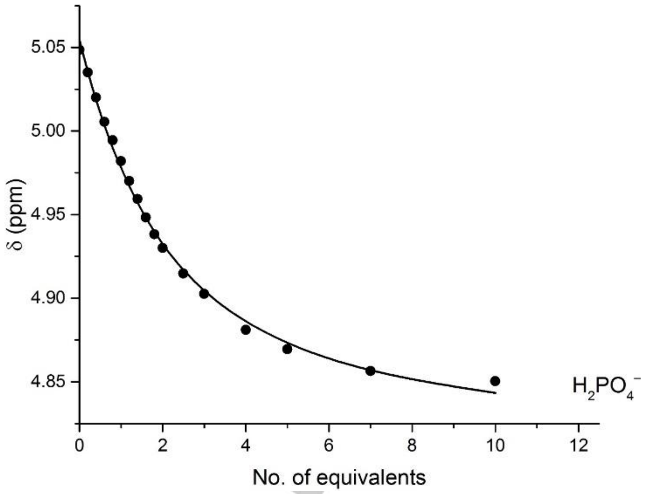

Figure S19. Titration curve for XB [3]rotaxane **5·(PF$_6$)$_2$** with dihydrogen phosphate. Curve obtained by monitoring the proton H$_o$ by ^{1}H NMR spectroscopy in (1:1 $CDCl_3$/CD_3OD, 298 K, 500 MHz). Solid points represent experimental data; continuous line represents theoretical binding isotherm.

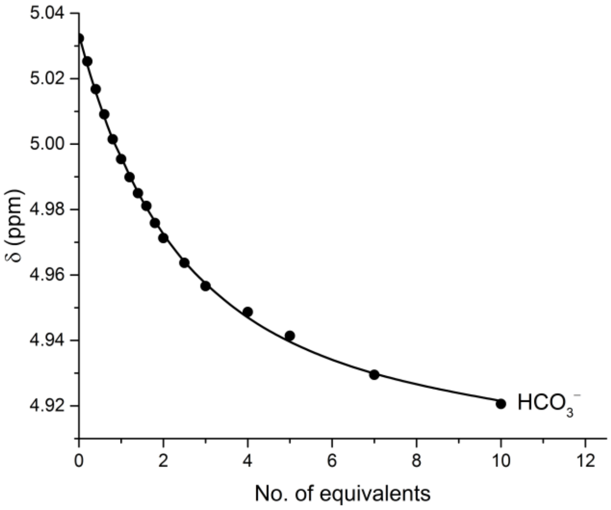

Figure S20. Titration curve for XB [3]rotaxane **5·(PF$_6$)$_2$** with hydrogen carbonate. Curve obtained by monitoring the proton H$_o$ by ^{1}H NMR spectroscopy in (1:1 $CDCl_3/CD_3OD$, 298 K, 500 MHz). Solid points represent experimental data; continuous line represents theoretical binding isotherm.

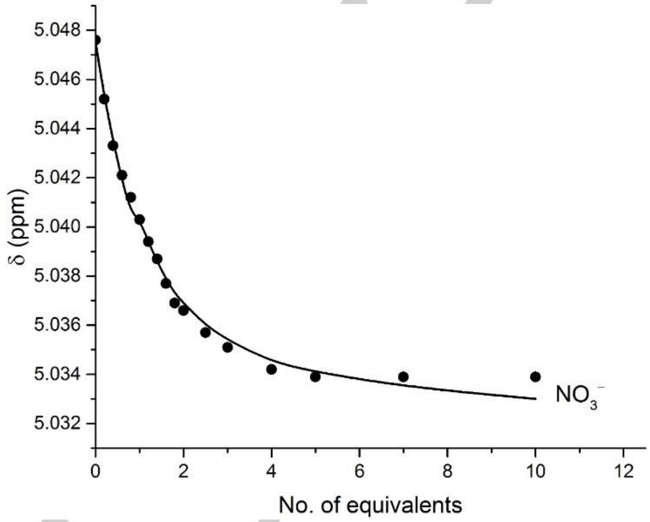

Figure S21. Titration curve for XB [3]rotaxane **5·(PF$_6$)$_2$** with nitrate. Curve obtained by monitoring the proton H$_o$ by ^{1}H NMR spectroscopy in (1:1 $CDCl_3/CD_3OD$, 298 K, 500 MHz). Solid points represent experimental data; continuous line represents theoretical binding isotherm.

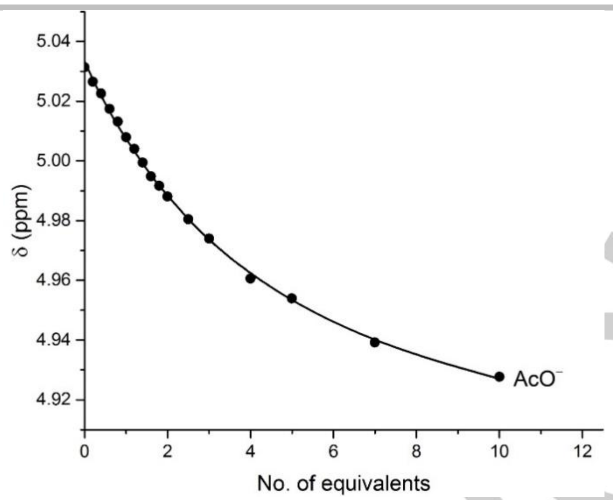

Figure S22. Titration curve for XB [3]rotaxane **5·(PF$_6$)$_2$** with acetate. Curve obtained by monitoring the proton H$_o$ by ^{1}H NMR spectroscopy in (1:1 $CDCl_3$/CD_3OD, 298 K, 500 MHz). Solid points represent experimental data; continuous line represents theoretical binding isotherm.

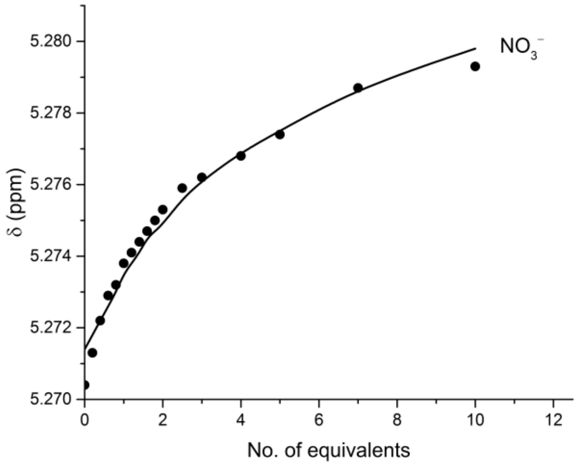

Figure S23. Titration curve for HB axle **1·(BF$_4$)$_2$** with nitrate. Curve obtained by monitoring the proton H$_o$ by ^{1}H NMR spectroscopy in (1:1 $CDCl_3$/CD_3OD, 298 K, 500 MHz). Solid points represent experimental data; continuous line represents theoretical binding isotherm.

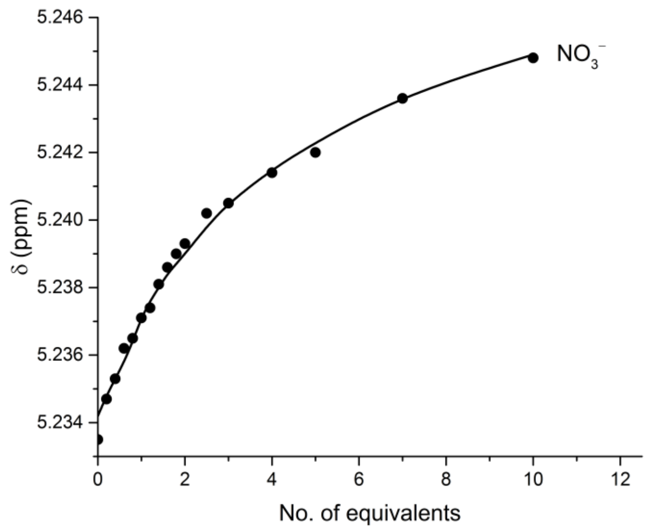

Figure S24. Titration curve for XB axle **2·(BF$_4$)$_2$** with nitrate. Curve obtained by monitoring the proton H_o by ^{1}H NMR spectroscopy in (1:1 $CDCl_3$/CD_3OD, 298 K, 500 MHz). Solid points represent experimental data; continuous line represents theoretical binding isotherm.

WILEY-VCH

Part V: Molecular Modelling Methods and Additional Data

Starting structure of XB [3]rotaxane

The structure of the macrocyclic components of **5** was taken from a previous work,[6] while the starting structure for the axle of **5** was generated via atomic manipulation of the crystal structure deposited with the Cambridge Crystallographic Data Centre (CCDC),[7] under RefCode ZEZDOZ.[8] The XB [3]rotaxane was obtained by the assembly of one axle and two macrocycle molecules in an interlocked fashion with the bis-iodo triazolium binding units and the macrocyclic clefts adopting an almost orthogonal relative disposition. This structure was further used to generate the **5·(Cl)₂** and **11·NO₃⁻** complexes for Molecular Mechanics (MM) and Molecular Dynamics (MD) study.

Quantum calculations

All quantum calculations were carried out with the Gaussian09 software,[9] and included the derivatisation of restrained electrostatic potential (RESP) charges for the [3]rotaxane's axle component, as well as the preliminary parameterisation of XB interactions on the chloride and nitrate complexes' models as detailed below.

Classical force field calculations

All MM and MD simulations were carried out with Amber14.[10] The rotaxane components (axle and macrocycle) were described with parameters taken from the Generalized Amber Force Field (GAFF)[11,12] and RESP charges.[13] The nitrate anion was described with bond terms force field parameters derived at the MP2/6–31+G* level, listed in Table S1 along with its RESP charges (*vide infra*). The van der Waals parameters were directly taken from GAFF. The chloride anions were described with a –1 discrete charge and van der Waals parameters developed for the TIP3P water model.[14] The all-atoms models of the methanol and chloroform solvent molecules were described with force field parameters and charges taken from refs. 19 and 20, respectively. The force field parameters and charges of the PF_6^- counter-ion were taken from ref. [17]. The post-processing of trajectory files to obtain the structural data was performed with *cpptraj*.[18]

Table S1. Force field parameters and RESP charges for nitrate.

Bond length	r_{eq} (Å)	K_r (kcal mol^{-1} Å^{-2})	
N–O	1.273	626.30	
Bond angle	θ_{eq} (º)	K_θ (kcal mol^{-1} rad^{-2})	
O–N–O	120.00	75.440	
Improper dihedral	Φ_0 (º)	K_Φ (kcal mol^{-1})	*n*
O–O–N–O	180	10.5	2
RESP charges	*q(e)*		
N	1.088883		
O	-0.696294		

WILEY-VCH

Parameterisation of XB interactions

The force field parameterisation of XB interactions was preceded by DFT optimisations of model complexes composed by a model axle incorporating the bis-iodo-triazolium motif (**8*Ph***) and chloride or nitrate anions. In 8***Ph***, the bulky stoppers, the naphthalene diimide stations and the biphenyl spacer moieties of the axle were replaced by phenyl rings. These preliminary quantum calculations were carried out using the B3LYP functional, with hydrogen, carbon, nitrogen, oxygen and chlorine atoms treated with the 6–311++G** basis set, while the iodine atoms were described with the aug-cc-pVDZ-PP basis set,[19,20] obtained from the EMSL website.[21,22] The optimised structures of **8*Ph*·Cl_2** and **8*Ph*·NO_3^-** are shown in Figure S12 and allowed us to ascertain, in gas-phase, the I···Cl^- distances of 2.638 Å and I···O distances of 2.592 Å, which guided the further parameterisation of the halogen bonding interactions.

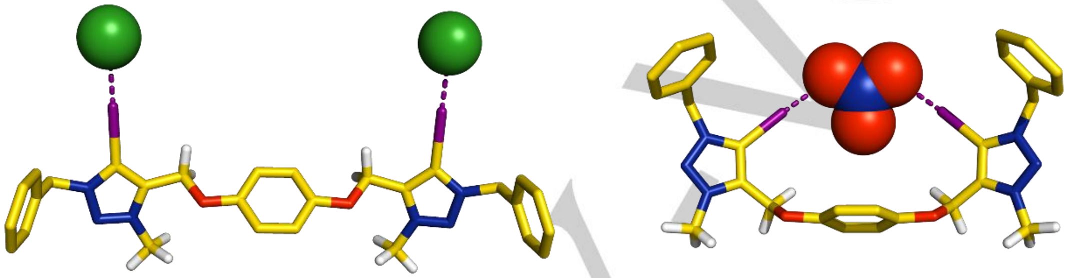

Figure S25. DFT optimised structures of **8*Ph*·Cl_2** (left) and **8*Ph*·NO_3^-** (right).

Likewise in our previous classical molecular dynamics investigations on anion recognition,[23–25] the halogen bonding interactions were represented by the addition of a massless extra-point (EP) of charge to GAFF, following the methodology established by Ibrahim.[26]

Several I–EP distances were systematically tested in gas phase *via* MM energy minimisations of the complexes **8*Ph*·Cl_2** and **8*Ph*·NO_3^-**, using RESP charges estimated for each I–EP distance. In a previously HF/6–31G* optimised structure of **8*Ph***, an extra point was positioned in front of each C–I bond at the desired I–EP distances and then the corresponding atomic charges were obtained *via* two stages RESP fitting (*vide infra*). The ranges of I–EP distances tested for the **8*Ph*·Cl_2** and **8*Ph*·NO_3^-** model complexes are gathered in Tables S2 and S3, respectively, together with MM I···Cl^- and I···O distances and the iodine and EP charges.

Table S2. Summary of MM optimised distances for **8*Ph*·Cl_2** as function of the I–EP distance, along with EP and iodine RESP charges.

Distances (Å)		Charges *q(e)*	
I–EP	I···Cl^-	EP	I
No EP	4.597 ; 4.597	–	0.296772
2.30	3.308 ; 3.308	0.079070	0.032879
2.31	3.304 ; 3.304	0.078178	0.034870
2.32	3.301 ; 3.301	0.077293	0.036854
2.33	3.297 ; 3.297	0.076417	0.038834
2.34	Atomic clash	0.075548	0.040808

WILEY-VCH

Table S3. Summary of MM optimised distances for $\mathbf{8}_{Ph}\cdot\mathbf{NO_3^-}$ as function of the I–EP distance along with EP and iodine RESP charges.

Distances (Å)		Charges *q(e)*	
I–EP	I···O	EP	I
No EP	4.806 ; 4.468	–	0.296772
2.04	2.852 ; 2.852	0.105502	-0.021343
2.05	2.844 ; 2.844	0.104353	-0.019149
2.06	2.833 ; 2.833	0.103217	-0.016966
2.07	2.821 ; 2.822	0.102092	-0.014792
2.08	2.810 ; 2.810	0.100978	-0.012629
2.09	2.796 ; 2.797	0.099876	-0.010475
2.10	2.778 ; 2.778	0.098786	-0.008331
2.11	2.752 ; 2.752	0.097706	-0.006196
2.12	2.711 ; 2.712	0.096637	-0.004069
2.13	Atomic clash	0.095578	-0.001952

Tables S2 and S3 show that several I–EP distances can be applied in the MM geometry optimisations of $\mathbf{8}_{Ph}\cdot\mathbf{Cl_2}$ or $\mathbf{8}_{Ph}\cdot\mathbf{NO_3^-}$. However, the subsequent use of the longer I–EP distances (2.31 to 2.33 Å for $\mathbf{8}_{Ph}\cdot\mathbf{Cl_2}$ and 2.05 to 2.12 Å for $\mathbf{8}_{Ph}\cdot\mathbf{NO_3^-}$) MD simulations of these complexes, carried out in gas phase at 300K for 5 ns, were shown to be instable, leading definitively to the exclusion of these distances. Therefore, these MD simulations were extended to shorter I–EP distances, and the 2.30 and 2.04 Å. These I–EP distances were further validated in gas phase MD simulations of pseudo [3]rotaxanes $\mathbf{5}_{Ph}\cdot\mathbf{Cl_2}$ and $\mathbf{5}_{Ph}\cdot\mathbf{NO_3^-}$, which were obtained by the assembly of two macrocycles with the $\mathbf{8}_{Ph}\cdot\mathbf{Cl_2}$ and $\mathbf{8}_{Ph}\cdot\mathbf{NO_3^-}$ complexes, respectively. For $\mathbf{5}_{Ph}\cdot\mathbf{Cl_2}$ I···Cl^- average distances of 3.391 ± 0.099 and 3.393 ± 0.098 Å were calculated, while for $\mathbf{5}_{Ph}\cdot\mathbf{NO_3^-}$, I···O average distances of 2.930 ± 0.104 and 2.919 ± 0.099 Å were assessed. Therefore, the 2.30 and 2.04 Å I–EP distances were appointed for the subsequent calculation of the final RESP charges of the capped bis-iodo triazolium anion recognition fragment.

Calculation of RESP charges on the XB [3]rotaxane

The RESP charges for the axle component were derived using a fragment-based approach due to its large size (318 atoms). Thus, two capped entities were built as sketched in Figure S13. The $\mathbf{8}_{stopper}$ fragment comprises the bulky stopper tris(*p*-tert-butylphenyl)(phenyl)methane, a naphthalene diimide station and a biphenyl spacer moiety capped with a methyl group. The $\mathbf{8}_{Ph}$ fragment incorporates the axle bis-iodo triazolium anion recognition site with the bulky stoppers, the naphthalene diimide stations and the biphenyl spacer moieties capped by phenyl substituents. These hypothetical molecules were optimised using the HF method coupled with 6–31G* basis set for all atoms, apart of the iodine atoms, which were treated with the aug-cc-pVDZ-PP basis set (*vide supra*). The RESP atomic charges were obtained in a two RESP charge fitting stages from the electrostatic potential estimated at the same level of theory using the Gaussian IOp: 6/33=2, 6/41=4, 6/42=6. Moreover, for $\mathbf{8}_{Ph}$ the following constraints were imposed*: a)* each methylene group (linking the central bis-iodo triazolium anion recognition site and aromatic capping groups – in red in Figure S13), must have the same net charge as the methyl substituent (in blue in Figure S13) removed from $\mathbf{8}_{stopper}$; *b)* the overall charge of the phenyl capping group and the

WILEY-VCH

methylene linker must amount to 0, while the net charge of the central bis-iodo triazolium anion recognition site (including the two extra-points) was set to 2.
After the charge calculation for **8***stopper* and **8***Ph*, their capping groups were removed, and the axle with RESP charges was generated attaching two capped **8***stopper* units to the methylene bridges of capped **8***Ph* central fragment.

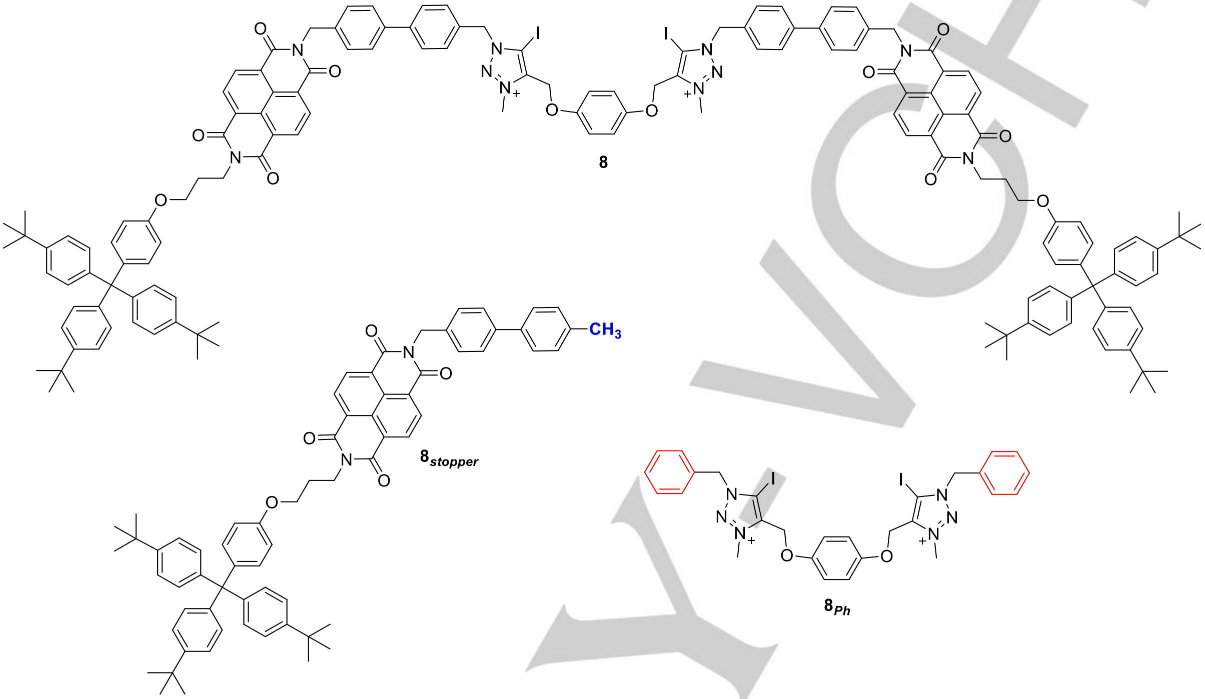

Figure S26. Capped entities used to derive the **8** axle RESP charges. The capping groups of **8***Ph* are identified in red and the capping group of **8***stopper* is identified in blue.

The RESP atomic charges for the macrocycle were the same as in our previous work.[6] The atomic charges for the nitrate anion given in Table S1 were also RESP charges determined from optimised at the HF/6–31+G* level using the number of layers and the number of points per layer quoted above.

General MD simulation methods

The starting geometries of **5·(Cl)₂** or **5·NO₃⁻** (*vide supra*) were minimised in gas phase by MM until the convergence criterion of 0.0001 kcal mol^{-1} was achieved. The MM optimised structures were then solvated in cubic boxes with 3387 chloroform molecules and 6690 methanol molecules in agreement with a 1:1 v/v solvent mixture used in ^{1}H NMR experimental binding studies. In addition, a PF_6^- counter-ion was added to solvated **5·NO₃⁻** to neutralise the system net charge. Each solvated system was equilibrated under periodic boundary conditions using the following multistage protocol. The system was relaxed by MM minimisation of solvent molecules and by keeping the solutes fixed with a positional restraint of 500 kcal mol^{-1} Å^{-2}. The restraint was then removed and the entire system was allowed to relax. Both minimisation stages comprised an initial set of 10000 steepest descent algorithm steps, followed by 10000 steps of conjugated gradient algorithm. The equilibration stage proceeded with heating up the system to 300 K for 100 ps using a NVT ensemble and a weak positional restraint (10 kcal mol^{-1} Å^{-2}) on the solutes. Afterwards, each system's density was allowed to equilibrate in a NPT ensemble at 1 atm for 1 ns, at the same temperature, followed by a NPT data collection run for 100 ns. The collection run's

WILEY-VCH

trajectory frames were saved every 1 ps. Three independent replicates were performed for each system. The CUDA version of the PMEMD executable was used for the simulation of all solvated systems.[27–29] The bond lengths involving all bonds to hydrogen atoms were constrained with the SHAKE algorithm allowing the usage of 2 fs time step.[30] The Particle Mesh Ewald (PME) method was used to treat the long-range electrostatic interactions.[31] The non-bonded van der Waals interactions were truncated with a 10 Å cut-off.

WILEY-VCH

Additional Data

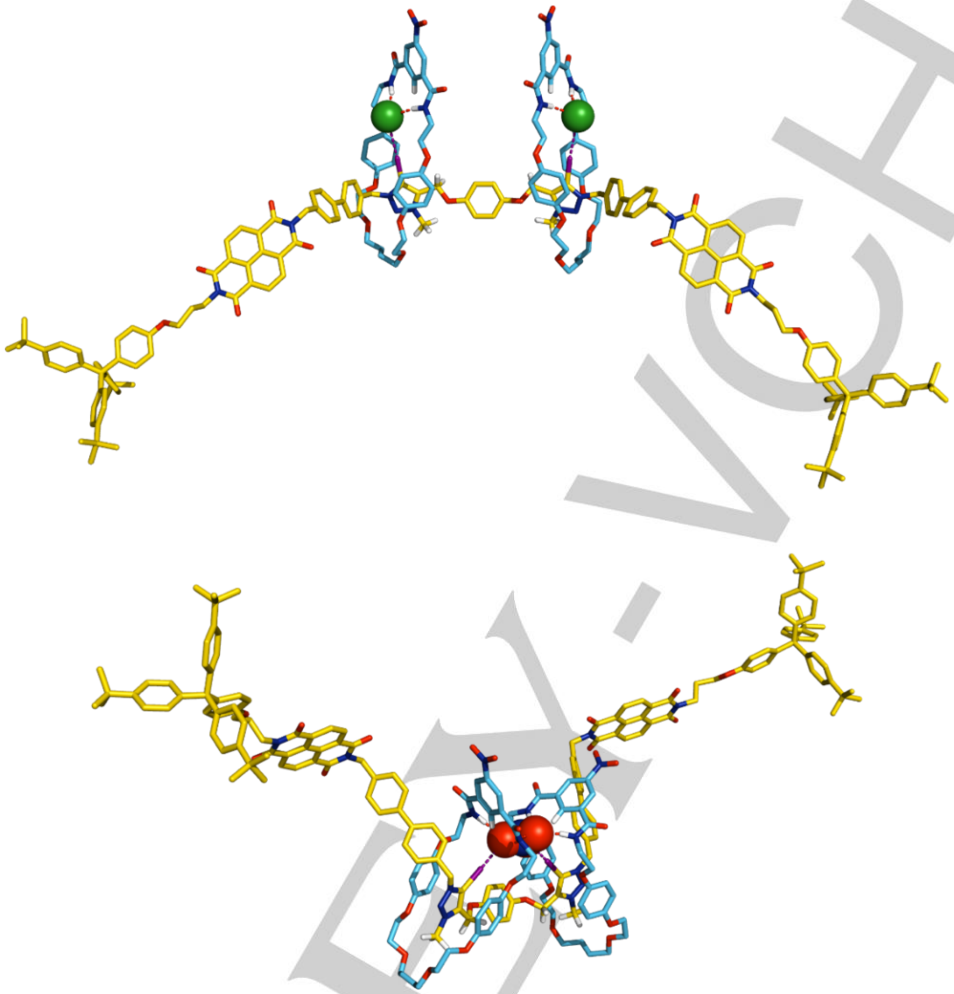

Figure S27. Starting anion [3]rotaxane binding scenarios of **5·(Cl)$_2$** (top) and **5·NO$_3^-$** (bottom) used in the MD simulations in $CHCl_3$:CH_3OH.

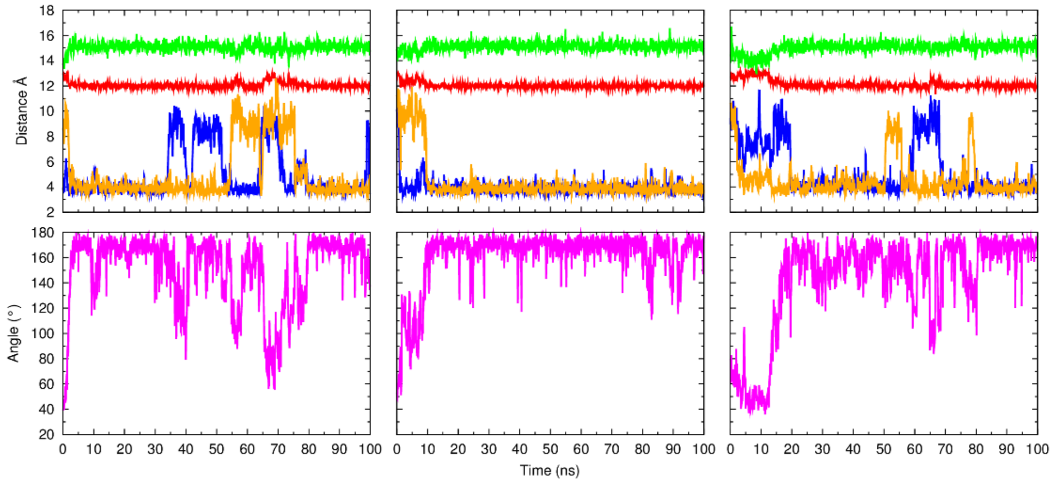

Figure S28. Evolution of relevant structural parameters throughout the 100 ns three independent MD runs of **5·(Cl)$_2$** (1 – left; 2 – centre; and 3 – right). Top: Cl$^-$···Cl$^-$ (green line) and I···I (red line) distances together with the two individual distances between the centroid of macrocyclic isophthalamide phenyl rings (rings ***1*** and ***2***) and the centroid of the axle's central hydroquinone phenyl ring (ring ***3***) runs (blue and orange lines); Bottom: ***1-3-2*** angle between phenyl rings' centroids.

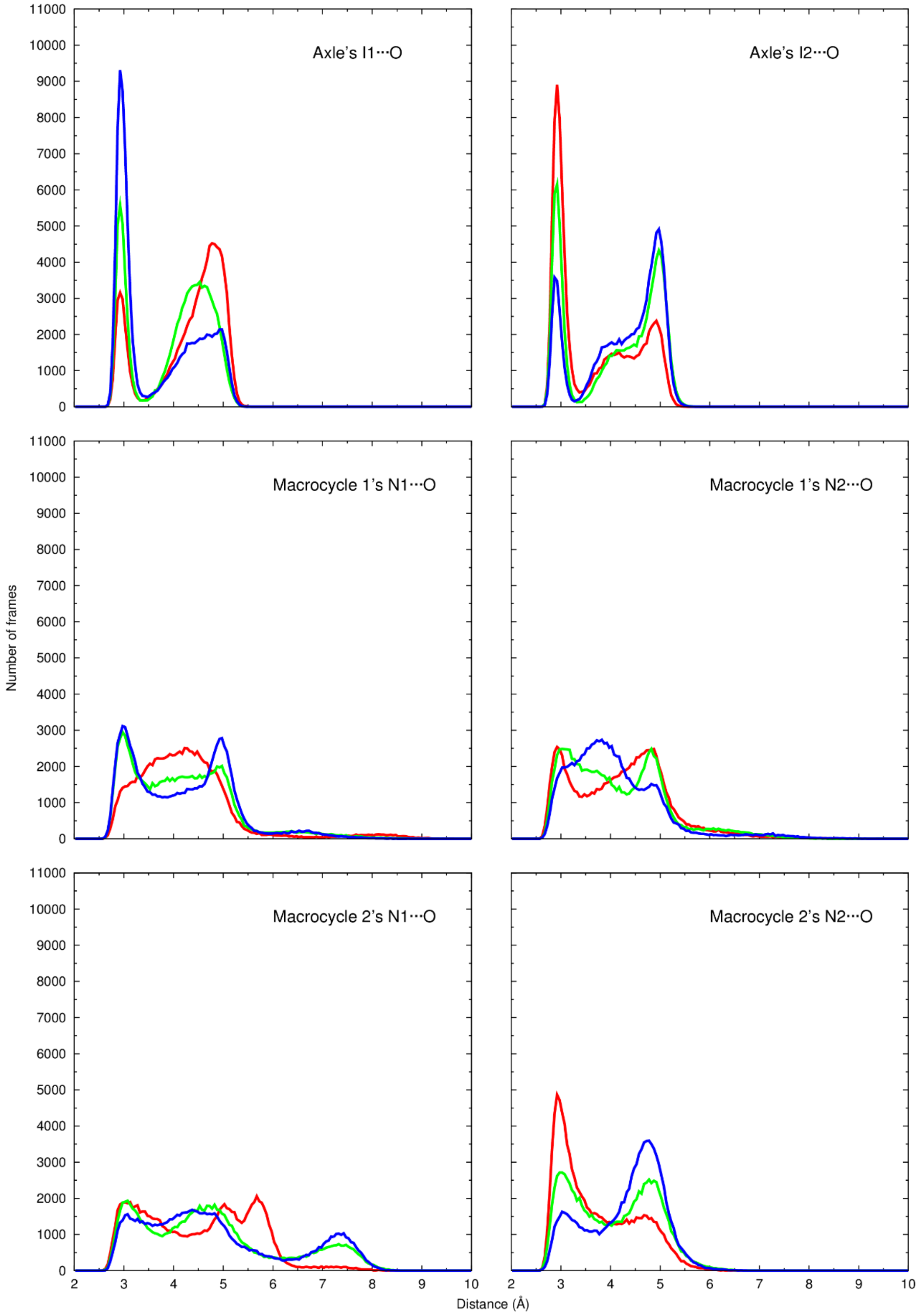

Figure S29. 1D histograms created from the I···O distances (top) and N···O distances (middle and bottom) observed along the 100 ns simulation time in MD run 1 of **5·NO$_3^-$**, for the independent binding units (axle's C–I or macrocycle's N–H) with the nitrate oxygen atoms (red, green and blue lines).

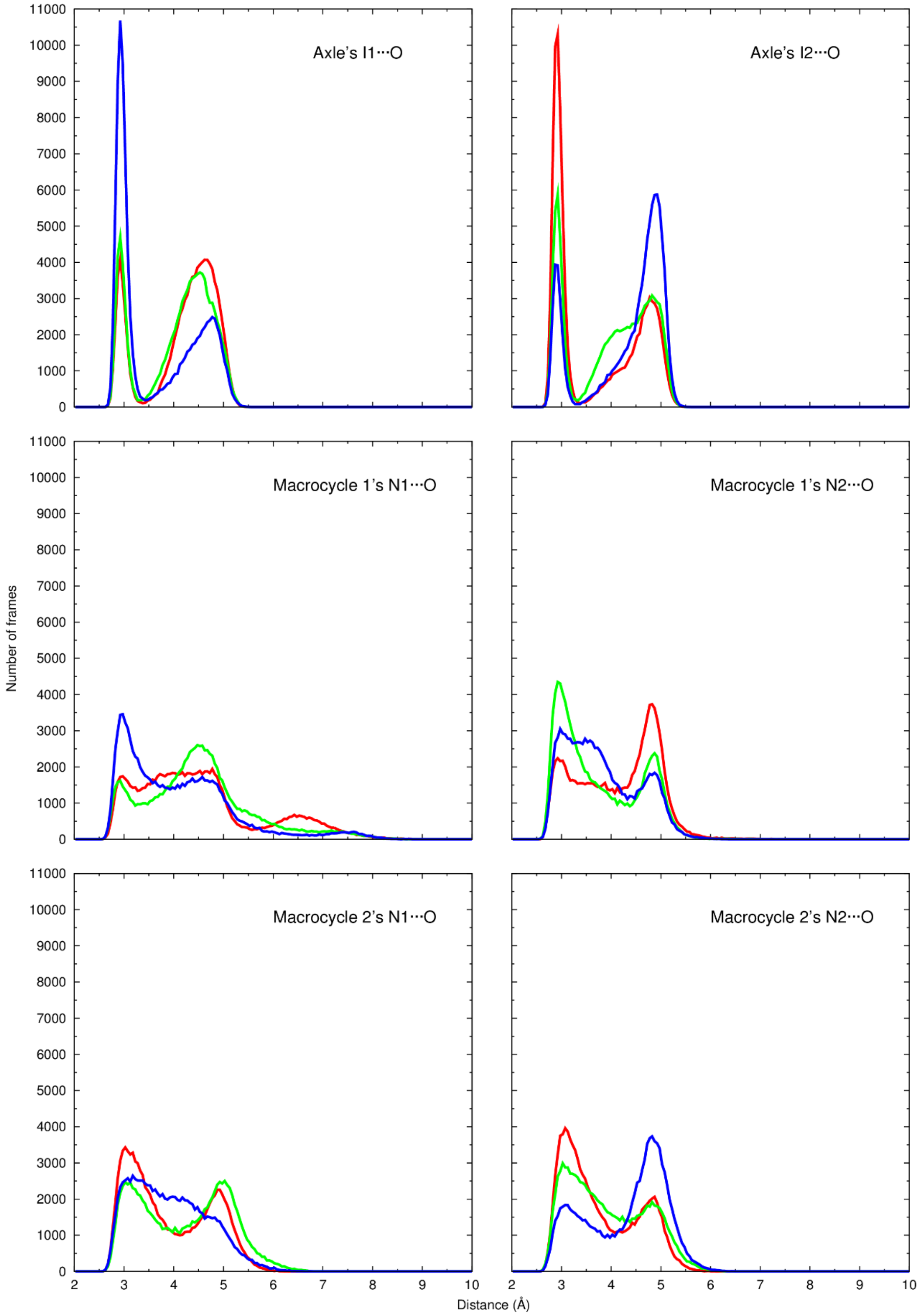

Figure S30. 1D histograms created from the I···O distances (top) and N···O distances (middle and bottom) observed along the 100 ns simulation time in MD run 2 of **5·NO$_3^-$**, for the independent binding units (axle's C–I or macrocycle's N–H) with the nitrate oxygen atoms (red, green and blue lines).

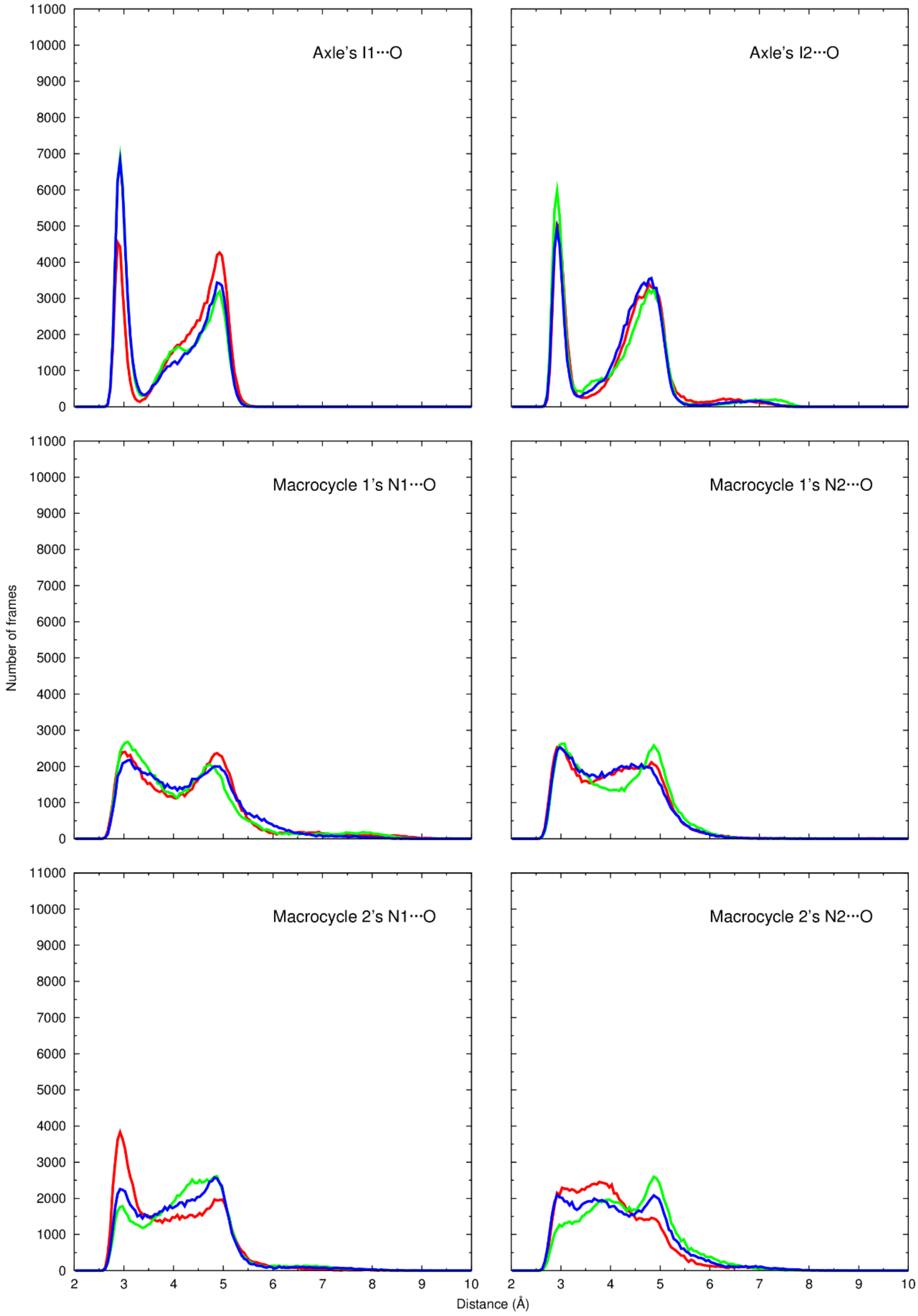

Figure S31. 1D histograms created from the I···O distances (top) and N···O distances (middle and bottom) observed along the 100 ns simulation time in MD run 3 of **5·NO$_3^-$**, for the independent binding units (axle's C–I or macrocycle's N–H) with the nitrate oxygen atoms (red, green and blue lines).

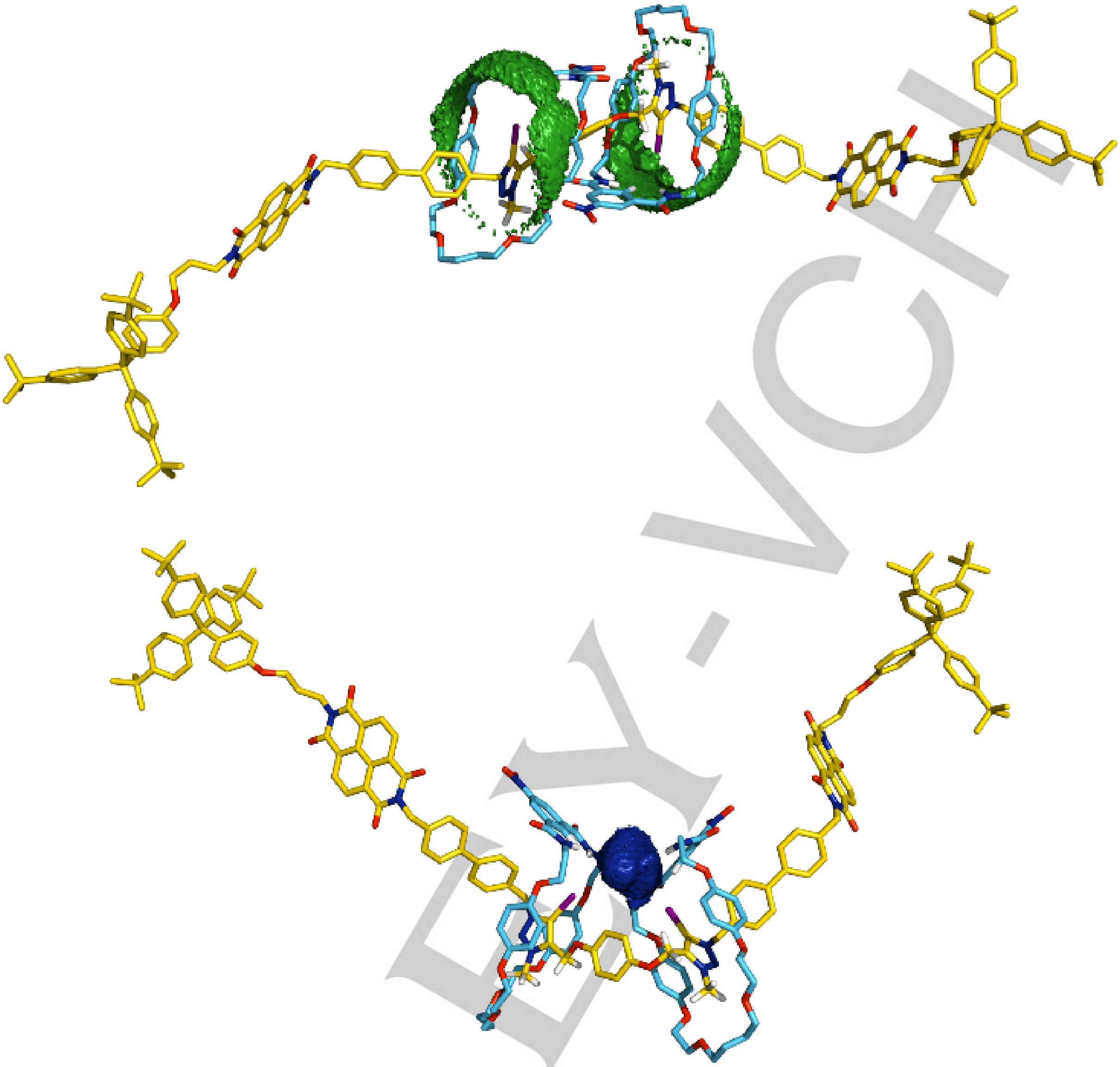

Figure S32. 3D histograms built with the positions occupied by the anions along one of the 100 ns MD simulation runs with **5·(Cl)$_2$** (top, green clouds) or **5·NO$_3^-$** (bottom, blue cloud).

WILEY-VCH

Table S4. Statistics for halogen bonding interactions evaluated throughout 100 ns in the individual MD simulation runs of **5·(Cl)$_2$** and **5·NO$_3^-$**.[a]

Run	Distances (Å)		Angles (°)	
	I⋯Cl$^-$	I⋯O	C–I⋯Cl$^-$	C–I⋯O
1	3.489 ± 0.134	2.969 ± 0.121	173.9 ± 3.4	174.5 ± 3.0
	3.490 ± 0.135	2.951 ± 0.121	173.9 ± 3.5	174.7 ± 3.0
2	3.492 ± 0.136	2.955 ± 0.115	174.0 ± 3.4	174.5 ± 3.0
	3.492 ± 0.136	2.932 ± 0.105	174.0 ± 3.4	175.2 ± 2.6
3	3.491 ± 0.137	2.956 ± 0.128	173.8 ± 3.7	174.6 ± 3.2
	3.489 ± 0.136	3.152 ± 0.585	173.8 ± 3.5	172.7 ± 6.9

[a] *N* = 100000

Table S5. Statistics for hydrogen bonding interactions evaluated throughout 100 ns in the individual MD simulation runs of **5·(Cl)$_2$** and **5·NO$_3^-$**.[a]

Run	Distances (Å)		Angles (°)	
	N⋯Cl$^-$	N⋯O	N–H⋯Cl$^-$	N–H⋯O
1	3.574 ± 0.229	3.373 ± 0.755	153.1 ± 12.5	153.1 ± 16.3
	3.571 ± 0.242	3.365 ± 0.631	155.2 ± 10.8	151.0 ± 18.5
	3.572 ± 0.230	3.826 ± 1.083	153.1 ± 12.0	128.0 ± 45.3
	3.568 ± 0.225	3.135 ± 0.309	154.8 ± 10.7	155.7 ± 14.7
2	3.591 ± 0.228	3.526 ± 0.875	151.4 ± 12.0	142.9 ± 26.4
	3.570 ± 0.220	3.100 ± 0.292	154.6 ± 9.5	153.5 ± 18.8
	3.592 ± 0.233	3.166 ± 0.293	151.1 ± 12.4	155.1 ± 17.1
	3.575 ± 0.231	3.153 ± 0.261	154.7 ± 9.7	159.7 ± 12.5
3	3.576 ± 0.261	3.400 ± 0.791	153.1 ± 12.4	152.5 ± 18.0
	3.579 ± 0.273	3.238 ± 0.454	154.6 ± 11.2	153.6 ± 18.3
	3.600 ± 0.279	3.273 ± 0.582	153.1 ± 11.5	152.9 ± 17.1
	3.586 ± 0.275	3.412 ± 0.585	153.7 ± 10.9	144.9 ± 22.8

[a] *N* = 100000

Supplementary MD Movies Captions

Movie S1. Movie of the second MD run of **5·(Cl)$_2$** (between the 1st and the 15th ns), showing the half-circumrotation conversion process from co-conformation ***A***, through ***B***, into co-conformation ***C***.

Movie S2. Movie of the first MD run of **5·NO$_3^-$** (between the 1st and the 100th ns), showing the stability of the anion association during the conversion between co-conformations ***A*** and ***B***.

WILEY-VCH

References

[1] B.-Y. Lee, S. R. Park, H. B. Jeon, K. S. Kim, *Tetrahedron Lett.* **2006**, *47*, 5105–5109.
[2] H. Zheng, W. Zhou, J. Lv, X. Yin, Y. Li, H. Liu, Y. Li, *Chem. – Eur. J.* **2009**, *15*, 13253–13262.
[3] L. M. Hancock, P. D. Beer, *Chem. Commun.* **2011**, *47*, 6012–6014.
[4] S. Erbas-Cakmak, E. U. Akkaya, *Angew. Chem. Int. Ed.* **2013**, *52*, 11364–11368.
[5] M. R. Sambrook, P. D. Beer, M. D. Lankshear, R. F. Ludlow, J. A. Wisner, *Org. Biomol. Chem.* **2006**, *4*, 1529–1538.
[6] N. G. White, A. R. Colaço, I. Marques, V. Félix, P. D. Beer, *Org. Biomol. Chem.* **2014**, *12*, 4924.
[7] F. H. Allen, *Acta Crystallogr. B* **2002**, *58*, 380–388.
[8] H.-P. Jacquot de Rouville, J. Iehl, C. J. Bruns, P. L. McGrier, M. Frasconi, A. A. Sarjeant, J. F. Stoddart, *Org. Lett.* **2012**, *14*, 5188–5191.
[9] M. J. Frisch, G. W. Trucks, H. B. Schlegel, G. E. Scuseria, M. A. Robb, J. R. Cheeseman, G. Scalmani, V. Barone, B. Mennucci, G. A. Petersson, et al., *Gaussian~09 Revision A.01*, **n.d.**
[10] D. A. Case, J. T. Berryman, R. M. Betz, D. S. Cerutti, I. T.E. Cheatham, T. A. Darden, R. E. Duke, T. J. Giese, H. Gohlke, A. W. Goetz, et al., *AMBER 2015*, University Of California, San Francisco, **2015**.
[11] J. Wang, R. M. Wolf, J. W. Caldwell, P. A. Kollman, D. A. Case, *J Comput Chem* **2004**, *25*, 1157–74.
[12] J. Wang, R. M. Wolf, J. W. Caldwell, P. A. Kollman, D. A. Case, *J. Comput. Chem.* **2005**, *26*, 114–114.
[13] C. I. Bayly, P. Cieplak, W. Cornell, P. A. Kollman, *J. Phys. Chem.* **1993**, *97*, 10269–10280.
[14] P. Li, L. F. Song, K. M. Merz, *J. Chem. Theory Comput.* **2015**, *11*, 1645–1657.
[15] T. Fox, P. A. Kollman, *J. Phys. Chem. B* **1998**, *102*, 8070–8079.
[16] J. W. Caldwell, P. A. Kollman, *J. Phys. Chem.* **1995**, *99*, 6208–6219.
[17] Z. Liu, S. Huang, W. Wang, *J. Phys. Chem. B* **2004**, *108*, 12978–12989.
[18] D. R. Roe, T. E. Cheatham, *J. Chem. Theory Comput.* **2013**, *9*, 3084–3095.
[19] K. A. Peterson, D. Figgen, E. Goll, H. Stoll, M. Dolg, *J. Chem. Phys.* **2003**, *119*, 11113.
[20] K. A. Peterson, B. C. Shepler, D. Figgen, H. Stoll, *J. Phys. Chem. A* **2006**, *110*, 13877–13883.
[21] D. Feller, *J. Comput. Chem.* **1996**, *17*, 1571–1586.
[22] K. L. Schuchardt, B. T. Didier, T. Elsethagen, L. Sun, V. Gurumoorthi, J. Chase, J. Li, T. L. Windus, *J. Chem. Inf. Model.* **2007**, *47*, 1045–1052.
[23] M. J. Langton, S. W. Robinson, I. Marques, V. Félix, P. D. Beer, *Nat. Chem.* **2014**, *6*, 1039–1043.
[24] M. J. Langton, I. Marques, S. W. Robinson, V. Félix, P. D. Beer, *Chem. - Eur. J.* **2016**, *22*, 185–192.
[25] J. Y. C. Lim, I. Marques, L. Ferreira, V. Félix, P. D. Beer, *Chem Commun* **2016**, DOI 10.1039/C6CC01701K.
[26] M. A. Ibrahim, *J Comput Chem* **2011**, *32*, 2564–74.
[27] A. W. Götz, M. J. Williamson, D. Xu, D. Poole, S. Le Grand, R. C. Walker, *J. Chem. Theory Comput.* **2012**, *8*, 1542–1555.
[28] R. Salomon-Ferrer, A. W. Götz, D. Poole, S. Le Grand, R. C. Walker, *J. Chem. Theory Comput.* **2013**, *9*, 3878–3888.

WILEY-VCH

[29] S. Le Grand, A. W. Götz, R. C. Walker, *Comput. Phys. Commun.* **2013**, *184*, 374–380.
[30] J.-P. Ryckaert, G. Ciccotti, H. J. . Berendsen, *J. Comput. Phys.* **1977**, *23*, 327–341.
[31] T. Darden, D. York, L. Pedersen, *J. Chem. Phys.* **1993**, *98*, 10089.
WILEY-VCH
